# Feeding the feelings: gender differences in emotional eating during COVID-19: a systematic review and meta-analysis

**DOI:** 10.3389/fnut.2025.1680872

**Published:** 2025-10-27

**Authors:** Christopher Zaiser, Marie Pahlenkemper, Gerrit Brandt, Cristina Ballero Reque, Luisa Sabel, Nora M. Laskowski, Georgios Paslakis

**Affiliations:** University Clinic for Psychosomatic Medicine and Psychotherapy, Medical Faculty, Campus East-Westphalia, Ruhr-University Bochum, Bochum, North Rhine-Westphalia, Germany

**Keywords:** COVID-19, coronavirus, emotional eating, gender differences, systematic review, health inequalities, meta-analysis, mental health

## Abstract

**Context:**

The COVID-19 pandemic intensified mental health issues and increased emotional eating (EE), a coping mechanism, where food is consumed in response to emotions rather than hunger. During the pandemic, gender-specific EE patterns were observed, with women reporting elevated EE levels in response to stress, anxiety, and depression due to various social and psychological factors.

**Objectives:**

This study primarily focused on examining gender differences in EE during the COVID-19 pandemic. As a secondary outcome, it aimed to explore predictors of EE.

**Data sources and extraction:**

This systematic review was pre-registered (PROSPERO CRD42023421727) and adhered to PRESS and PRISMA guidelines. Studies published between March 2020 and August 2024 were identified across Scopus, Web of Science, PubMed, and PsycINFO. The quality assessment was performed using the “Critical Appraisal Checklist for Analytical Cross-Sectional Studies.” The meta-analysis was conducted following MOOSE guidelines.

**Data analysis:**

Of 14,347 studies identified, 30 met inclusion criteria (only if population ≥18 years, without clinical diagnoses, gender-specific analysis regarding EE, observational studies with original data collection during COVID-19 pandemic), with 16 incorporated into the meta-analysis. Gender significantly moderated pandemic-related stress. Higher EE scores in women were linked to isolation and caregiving responsibilities, while men’s EE often appeared as reward-seeking. Across diverse measures and regions, women consistently exhibited higher EE scores (Cohen’s *d* = 0.39). Young adults and students showed a stronger association with EE, suggesting heightened vulnerability. Key predictors included increased food intake, COVID-19-related stress and lifestyle changes, sleep quality, and physical activity.

**Conclusion:**

The predominance of cross-sectional designs limits the ability to draw causal conclusions, and selection bias in studies, often targeting specific groups, restricts generalizability. Future longitudinal studies are needed to assess causality and explore the inferences to additional factors, such as socioeconomic status and mental health. Gender-sensitive interventions are suggested to address EE risks, particularly in women.

**Systematic review registration:**

PROSPERO (CRD42023421727). https://www.crd.york.ac.uk/prospero/display_record.php?ID=CRD42023421727.

## 1 Introduction

In March 2020, the World Health Organization officially recognized the COVID-19 outbreak as a global pandemic (1), which led to the widespread implementation of various safety measures, including lockdowns, social distancing mandates, and home confinements (2). These measures disrupted daily routines and lifestyles (3) and posed significant economic challenges for many individuals (4). Extended lockdowns and prolonged uncertainty significantly increased global stress and anxiety levels (5), intensifying existing mental health challenges, including disordered eating behaviors (DEB) and emotional eating (EE) (6, 7).

### 1.1 Emotional eating

EE has been defined as the tendency to consume food in response to emotions, both positive and negative, rather than to satisfy physical hunger (8). Given the societal health risks associated with obesity, the link between EE and excess weight underscores the importance of addressing this behavior as a public health issue (9, 10). EE is strongly connected with other DEB, such as loss of control over eating, binge-eating, and food cravings (11, 12), which also often lead to obesity (13).

Research shows that individuals prone to EE have distinct dietary patterns compared to non-emotional eaters, including higher consumption of sweet and high-fat meals (14) and more frequent snacking (15). Many individuals also experience low levels of interoceptive awareness (16), leading to difficulties in identifying hunger, satiety, and emotional states (17). This reduced awareness is often linked with alexithymia, a condition characterized by difficulties in recognizing and expressing emotions (18).

EE may function as a form of escapism from negative emotions (19), given the stress-relieving effect of consuming energy-dense foods that activate reward pathways and release dopamine (20). The EE pattern creates a feedback loop in which EE provides temporary relief from distressing emotions but may lead to feelings of shame and guilt when the individuals reflect on their past behavior, which reinforces the negative emotions and may thus further perpetuate EE (19, 21). Research indicates that certain emotional states—such as anxiety, sadness, tension, and boredom—are more likely to result in EE than other negative emotions (22, 23). Accordingly, an experimental study found that anxiety, but not anger, is associated with greater food consumption (24). Furthermore, EE has been shown to be highly correlated with perceived stress (25). Increased stress levels are associated with a reduced ability to accurately perceive internal signals of hunger and satiety, along with alterations in the body’s natural hunger cues (26).

### 1.2 Eating behaviors in the pandemic context

Limited access to healthcare services during the COVID pandemic exacerbated anxiety and affected treatment compliance, particularly impacting individuals with ongoing health needs (27). Stress associated with confinement was further linked to sleep disturbances, which, in turn, were associated with higher food consumption (28). The prolonged lockdowns and restrictions during the pandemic intensified social isolation and heightened levels of stress, anxiety, and fear (29). This has led to an increase in the consumption of high-caloric “comfort foods” as a coping mechanism for negative emotions (30). Continuous media exposure to COVID-19-related news also acted as a chronic stressor, further fueling overeating behaviors (31). Additional pandemic-driven changes, such as disruptions to physical activity routines and increased preoccupation with weight and body image, exacerbated DEB (27, 32–34). Notably, these pandemic-related stressors appear to have impacted DEB in a gender-specific way; the systematic analysis by Laskowski et al. (35) found that women exhibited higher levels of disordered eating compared to men.

### 1.3 Gender differences in emotional eating

Research highlights that stress responses differ by gender, with women showing a greater propensity for stress-induced EE (36). Studies consistently demonstrate that EE levels are higher in women than in men (37, 38). In particular, women more often report eating as a response to emotional states such as depression, anxiety, or anger, especially during periods of elevated stress (26, 37, 39, 40). This pattern suggests that women may be more inclined to rely on EE as a coping mechanism during times of emotional distress (41).

A contributing factor to the observed gender disparity in EE is its association with depressive symptoms, which are more commonly reported by women (42–44). The higher prevalence of depressive symptoms may make women more likely to engage in EE as a means of managing their emotional distress. This aligns with patterns observed in other DEB, such as binge eating (45), where women are also overrepresented (46). Research suggests that men and women often differ in their ability to identify and express emotions, potentially leading to higher self-reported EE in women (37, 47).

Pandemic-induced stress and its impact on emotional regulation also appear to have differed between men and women. Evidence suggests that women are more likely to use self-distraction, including EE, as a coping strategy in response to heightened stressors (48, 49). This gender-specific vulnerability reflects a combination of psychological (50), social (51), and biological factors (52, 53) that influence stress processing and emotional responses. Current research suggests differences in hypothalamic-pituitary-adrenal (HPA) axis activation (54), immune function (55) and brain regions associated with emotional processing (53). In addition, women reported higher levels of rumination during negative affect (56, 57), which can exacerbate feelings of loss of control, leading to increased depressive and anxious symptoms (58).

The persistent stressors of the pandemic—including caregiving responsibilities, work-life balance challenges, and frequent exposure to anxiety-provoking media—may have exacerbated these tendencies in women (21). Prolonged isolation, increased anxiety, and constant exposure to distressing news may amplify negative emotions and disrupt emotion regulation, potentially leading to more pronounced gender-specific eating responses (59–61). This highlights the complex dynamics between stress processing, emotional regulation (62), and societal pressures that differentially affect genders (62, 63).

Although several reviews have examined eating behaviors during the COVID-19 pandemic, there is a lack of systematic reviews with meta-analytic synthesis focusing specifically on gender differences in EE. Previous reviews either addressed broader DEB or reported gender as a secondary outcome, leaving the precise magnitude and moderators of gender-specific EE during the pandemic insufficiently clarified. Despite several reviews on eating behaviors during COVID-19, a quantitative synthesis focusing specifically on gender differences in emotional eating is thus still lacking.

### 1.4 Objectives of the study

Evidence indicates that EE is shaped by gender-specific patterns and the distinct stressors introduced by the COVID-19 pandemic (64, 65), highlighting the need for an in-depth investigation into these dynamics. This systematic review and meta-analysis aimed to synthesize evidence on gender differences in EE during the COVID-19 pandemic. Specifically, we sought to quantify effect sizes across studies to provide a precise estimate of gender-specific patterns and to explore potential moderators of these differences. By clarifying the magnitude and consistency of gender differences in EE, this study addresses a gap in the literature and provides evidence relevant for designing gender-sensitive interventions.

## 2 Methods

### 2.1 Criteria for inclusion and exclusion

We included original research articles written in English or German, with no geographical limitations. Eligible studies focused on the general adult population, regardless of gender, while studies involving children, adolescents (<18 years), or clinical populations were excluded. Studies were required to address outcomes explicitly related to EE with a clear gender-specific analysis. Publications before March 2020 and those written in languages other than English or German, were excluded. Research on general eating behavior or emotion regulation without a specific focus on EE, as well as studies lacking gender comparisons, were excluded. Excluded publication types comprised (systematic) reviews, meta-analyses, case reports, qualitative studies, commentaries, conference papers, opinion pieces, letters, and editorials. The inclusion and exclusion criteria according to the population, intervention, comparison, outcomes, study design (PICOS) scheme are shown in Table 1.

**Table 1:** PICOS criteria.

Criterion	Description
Population (P)	General adult population (≥18 years) without clinical diagnoses
Intervention (I)	No intervention
Comparison (C)	Gender-specific analysis
Outcomes (O)	Emotional eating
Study Design (S)	Quantitative observational studies with original data, data collection during COVID-19 pandemic

During screening, we verified that all included studies reported at least one measurement collected during the COVID-19 pandemic (March 2020 onward). Studies that also contained pre-pandemic data were eligible only if pandemic-period data were available and analyzed.

### 2.2 Literature search

This review was preregistered with the National Institute for Health and Care Research on the “Prospero International Prospective Register of Systematic Reviews” (registration number: CRD42023421727). No additional protocol document beyond the PROSPERO registration form was developed or published. An evidence-based electronic search was conducted following the “Peer Review of Electronic Search Strategies” (PRESS) guidelines to guarantee a rigorous and reproducible approach. Additionally, the review adhered to the “Preferred Reporting Items for Systematic Reviews and Meta-Analyses” (PRISMA 2020) guidelines (66) (see Supplementary material for checklist), ensuring a transparent, structured, and comprehensive reporting process.

A comprehensive search of the literature was performed across four major databases—Scopus, Web of Science (WOS), PubMed MEDLINE, and PsycINFO—covering the period from March 2020 to August 8, 2024. The initial search was conducted on July 13, 2023, and updated and finalized on August 8, 2024. Although the initial search syntax formally included records from 2019 onwards, only studies published from March 2020 onwards and reporting data collected during the COVID-19 pandemic were eligible for inclusion. The search query used for PubMed is displayed in Table 2, with search terms adapted as needed for each database. The complete search strategy for all databases is available at https://www.crd.york.ac.uk/PROSPEROFILES/421727_STRATEGY_20230713.pdf. The Covidence software (67) was employed to organize and manage the studies, including deduplication, title/abstract screening, and full-text review. We did not systematically search the grey literature, which may have limited the inclusion of non-indexed studies.

**Table 2:** Full search string for PubMed.

Topic	MeSH term
Eating behavior, body image	((diet[Title/Abstract]) OR (nutrition[Title/Abstract]) OR (binge*[Title/Abstract]) OR (eating behavio*[Title/Abstract]) OR (eating habit*[Title/Abstract]) OR (eating disorder*[Title/Abstract]) OR (disordered eating[Title/Abstract]) OR (body *satisfaction[Title/Abstract]) OR (body image[Title/Abstract])) AND
Gender	((gender*[Title/Abstract]) OR (sex[Title/Abstract]) OR (men[Title/Abstract]) OR (women[Title/Abstract]) OR (trans*[Title/Abstract]) OR (lgb[Title/Abstract]) OR (lgbt*[Title/Abstract]) OR (intersex*[Title/Abstract]) OR (*binary[Title/Abstract]) OR (queer[Title/Abstract]) OR (male[Title/Abstract]) OR (female[Title/Abstract])) AND
COVID-19	((sarscov[Title/Abstract]) OR (pandem*[Title/Abstract]) OR (COVID-19[Title/Abstract]) OR (corona*[Title/Abstract]) OR (lockdown[Title/Abstract]) AND
Language	((English[Language]) OR (German[Language])) AND
Time	(2019:2023[pdat]))

### 2.3 Study evaluation

A two-stage procedure was employed for study selection. In the first stage, titles and abstracts were screened independently by two reviewers, with assignments rotated among six investigators (CZ, MP, GB, CR, LS, and NL). In the second stage, two reviewers independently examined each full text using the same assignment procedure, with particular attention to whether questionnaires assessed and reported EE. If the two reviewers agreed, the decision was accepted; in case of disagreement, consensus was sought through discussion with the broader review team, and unresolved cases were adjudicated by the first author (CZ), in consultation with the senior authors (NL and GP) if needed. The final set of included studies was verified by the senior authors (NL and GP). The synthesis of outcomes was conducted by the first (CZ) and second (MP) author, and NL, who provided methodological oversight.

The quality assessment was performed using the “Critical Appraisal Checklist for Analytical Cross-Sectional Studies” developed by the Joanna Briggs Institute (68), following recommendations by Ma et al. (69). The checklist comprises eight items evaluated as “Yes,” “No,” “Unclear,” or “Not Applicable,” with an overall appraisal categorized as “Include,” “Exclude,” “Seek Further Information,” and “Comments.” Two authors independently conducted the quality assessment of included studies, with CZ making the final decision on assessments.

### 2.4 Statistical methods for meta-analysis

All statistical analyses were performed using the open-source software R version 4.4.1 (70), with the *metafor* package version 4.6.0 (71) employed to calculate Cohen’s *d*, perform the sensitivity analysis, and conduct subgroup analyses. In accordance with Cochrane’s recommendations for observational data, we followed the MOOSE guidelines (see Supplementary material for checklist) for conducting and reporting this meta-analysis of observational studies (72). In this meta-analysis, Cohen’s *d* was calculated as the standardized mean difference effect size based on mean values (M) and standard deviations (SD), either directly extracted from the included studies (*n* = 9) or obtained by contacting the corresponding authors (*n* = 21) to request (additional) data. These inquiries were conducted between September, 15th 2024 and October, 18th 2024. If no response was received, additional co-authors of the respective articles were contacted. If no extractable statistics were obtained after these attempts, the study was retained for the qualitative synthesis but excluded from the meta-analysis. The data associated with this study can be requested from the corresponding author.

We calculated the overall effect size and created a forest plot to visually summarize the findings across studies. To assess the robustness of our results, we performed a sensitivity analysis using the “leave-one-out” method, systematically removing one study at a time that had the greatest impact on heterogeneity, and recalculating the effect size for each iteration.

To gain deeper insights and identify potential moderators contributing to the observed variability, we conducted subgroup analyses targeting specific factors. The following data was extracted for this purpose: (1) measurement instruments: Emotional Eater Questionnaire (EEQ) (73), Emotional Eating Scale (EES) (8), Three-Factor-Eating Questionnaire (TFEQ) (74, 75), Eating Behavior Pattern Questionnaire (EBPQ) (76), Dutch Eating Behavior Questionnaire (DEBQ) (77), Emotional Overeating Questionnaire (EOQ-5) (78), self-developed; (2) percentage of women (<50%, 50–70, >70%); and (3) sample characteristics (young adults and university students, general population, and professionals). Finally, we examined (4) geographical regions, including Middle East, Europe, Latin America, South Asia, East Asia, cross-regional, and cross-regional Spanish-speaking. In this context, the “cross-regional” category includes one (36) study that evaluated participants from several different regions, while “cross-regional Spanish-speaking” refers to a study (79) that specifically included multiple Spanish-speaking countries across regions.

To assess heterogeneity, we calculated the *I*^2^ statistic and Cochran’s *Q*, with a significant *p*-value for Cochran’s *Q* indicating heterogeneity beyond chance. Publication bias was evaluated using Egger’s test and a funnel plot; a significant *p*-value for Egger’s test would suggest asymmetry, indicating potential publication bias. We applied a random-effects model to account for variability across studies, representing effect sizes as Cohen’s *d*. Forest plots were used to display both individual and overall Cohen’s *d* values.

## 3 Results

### 3.1 Extracted studies

**Figure 1:**
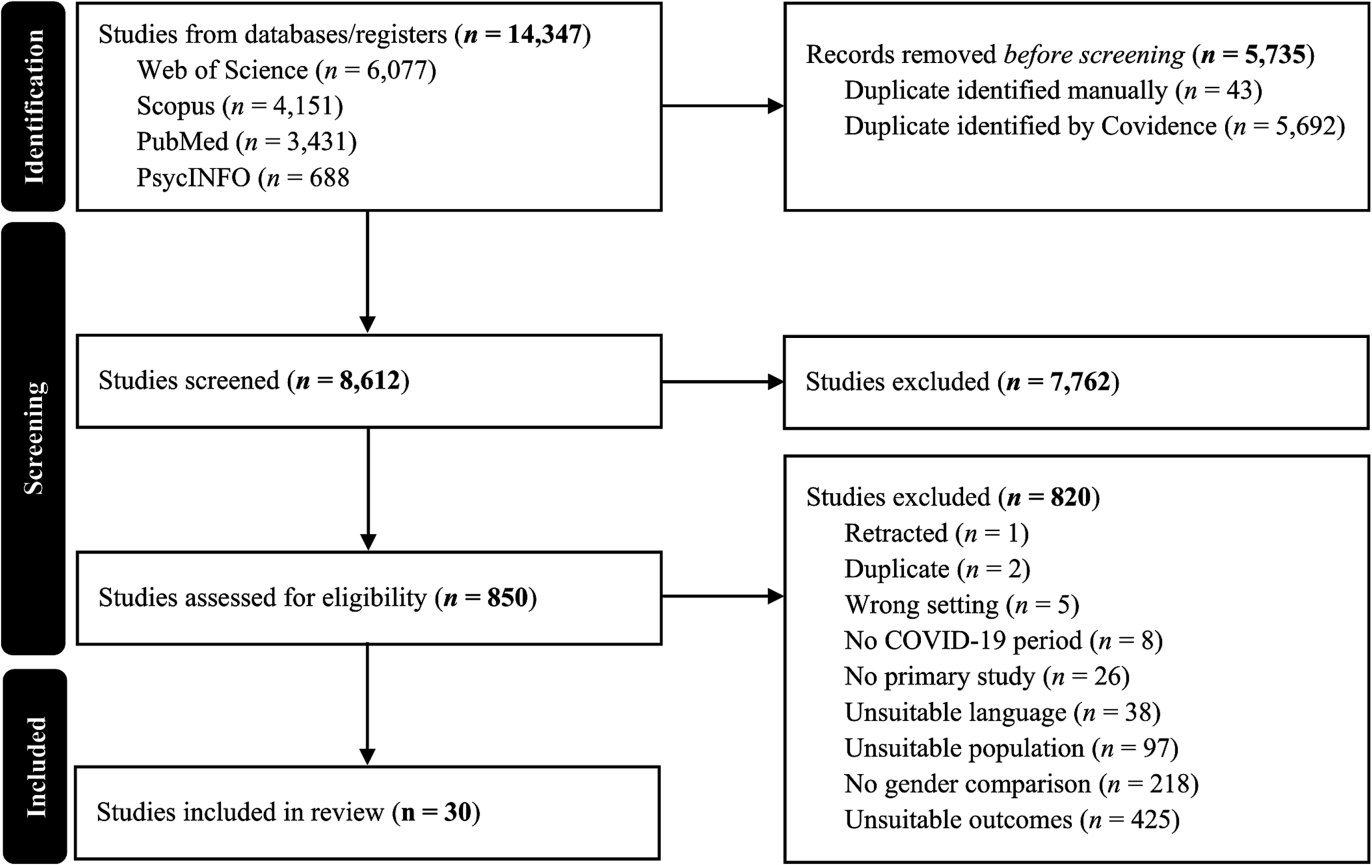
PRISMA diagram.

The literature search yielded 14,347 records. Following the selection process, 30 studies met the inclusion criteria and were included in the final synthesis (see PRISMA diagram, Figure 1). Of these, 16 studies were included in the meta-analysis. Studies entered the meta-analysis when they reported gender-disaggregated means and standard deviations (or convertible statistics) for emotional eating outcomes during the pandemic. The remaining studies (*n* = 14) did not provide sufficient quantitative information and were therefore retained in the narrative synthesis only.

### 3.2 Results regarding emotional eating

Thirty studies examining EE (see Table 3) were included in this review. Among these studies, 27 reported binary categories of women and men, while three also included “other” and “not specified” genders. The total sample size across all studies was 73,812 participants, comprising 46,935 women (63.6%), 26,830 men (36.3%), and 47 individuals (0.1%) classified as “other” or “not specified.” However, none of the studies provided detailed analyses for the “other” and “not specified” gender categories. Sample sizes in individual studies ranged from 120 to 24,968 participants.

**Table 3:** Gender differences in emotional eating during the COVID-19 pandemic.

References	Measure	Sample, country	Result gender difference
Barcln-Güzeldere and Devrim-Lanpir (123)^a^	EEQ	***N* = 506** (387 women, 119 men), Turkey	Women: M = 11.17 ± 5.85, men: M = 9.39 ± 5.37 ➔ sig. difference (*p* < 0.001)
Bemanian et al. (64)^a^	Self-developed	***N* = 24,968** (13,944 women, 11,024 men), Norway	EE past week: 62% of women, 43% of men More than 4 times of EE in the last week: 16% of women, 9% of men Gender as predictor of EE: OR = 1.9 [CI (1.8; 2.0)] ➔ higher chance of reporting EE in women compared to men
Carpio-Arias et al. (83)^a^	EEQ	***N* = 8,426** (5,943 women, 2,483 men), Ecuador (young adults)	Women: M = 8.04 ± 5.83, non-emotional eaters 40.6%, individuals with low EE 31.3%, EE 24.1%, individuals with high EE 4.0% Men: M = 6.31 ± 5.04, non-emotional eaters 51.6%, individuals with low EE 31.3%, EE 15.5%, individuals with high EE 1.6% EE behavior was sig. associated with gender [*F* (1)=186.67, *p* < 0.001]
Carpio-Arias et al. (90)	EES	***N* = 2,333** (1,581 women, 752 men), Ecuador	Gender [*F* (1) = 1.9, *p* = 0.16] did not show a sig. relationship with EE scores
De Diego et al. (92)	EE score was created by items associated with overeating	***N* = 2,084** (1,292 women, 792 men), Spain (workers from National Research Council)	33% of women vs. 25% of men reported that emotional changes had a negative impact on their eating habits (*p* < 0.001) Women selected EE-related statements more often than men: item “emotional appetite loss”: 10.1% vs. 4.8% (*p* < 0.001); item “loss of control with hyperpalatable foods”: 20.1% vs. 14.5% (*p* < 0.01); item “anxiety-/boredom-induced eating”: 31.7% vs. 27.0% (*p* < 0.05)
Du et al. (36)^a^	TFEQ-18	***N* = 1,392** (973 women, 379 men, 40 “other” gender), China (7.8%), Ghana (9.2%), Ireland (11.0%), Malaysia (7.3%), South Korea (7.5%), Netherlands (6.8%), United States of America (50.4%) (university students)	Women: M = 36.8 ± 22.3, men: M = 26.9 ± 21.7 ➔ sig. difference (*p* < 0.001) Women: Sig. correlation between EE and dietary risk (*B* = 0.024, *p* < 0.001) and between EE and BMI (*B* = 0.012, *p* = 0.043), no sig. correlation between EE and perceived stress (*B* = 0.443, *p* = 0.142) EE sig. mediated the relationship between perceived stress and dietary risk (*B* = 0.001, 95% CI = 0.007, 0.022) and the relationship between perceived stress and BMI (*B* = 0.024, 95% CI = 0.012, 0.041) Sig. interaction effect of sleep quality and EE on dietary risk (*p* = 0.022) and of sleep quality and EE on BMI (*p* = 0.015) ➔ better sleep quality weakens the relationship between EE and dietary risk and between EE and BMI Men: Sig. correlation between EE and perceived stress (*B* = 1.205, *p* < 0.001), no sig. correlation between EE and dietary risk (*B* = −0.011, *p* = 0.547) EE sig. mediated the relationship between perceived stress and BMI (*B* = 0.038, 95% CI = 0.014, 0.069) but not the relationship between perceived stress and dietary risk (*B* = 0.006, 95% CI = −0.005, 0.018) No sig. interaction effect of sleep quality and EE on dietary risk (*p* = 0.251), and of sleep quality and EE on BMI (*p* = 0.999)
Ederer et al. (95)	1 item (how often someone is taking comfort by eating in response to loneliness or stress during the COVID-19 pandemic)	***N* = 4,068** (2,099 women, 1,969 men), United States of America	Sig. association between gender and the following categories (*p* < 0.001): Never/rarely: 55.4% women vs. 73.4% men Sometimes: 31.6% women vs. 20.4% men Often/always: 12.9% women vs. 6.1% men
Elmacloǧlu et al. (98)	TFEQ-18	***N* = 1,036** (827 women, 209 men), Türkiye	No sig. difference in EE scores between women and men (*p* = 0.12)
Eşer Durmaz et al. (89)^a^	EES	***N* = 1,000** (792 women, 208 men), Türkiye (university students)	Women who spent >2 h/day on social media had higher scores compared to those who spent ≤2 h/day (*p* < 0.01); difference not sig. in men (*p* = 0.183)
Göbel et al. (87)	EES	***N* = 458** (309 women, 149 men), Türkiye	No EE tendency: 142 (58%) women vs. 103 (42%) men EE tendency: 167 (78.4%) women vs. 46 (21.6%) men ➔ sig. relationship between EE category and gender (*x*^2^ = 21.70, *p* < 0.001)
Hadar-Shoval et al. (65)	DEBQ	***N* = 1,969** (1,085 women, 884 men), Israel	Sig. correlation between EE and gender (*r* = 0.13, *p* < 0.001)
Hamzaid et al. (84)	EEQ	***N* = 210** (154 women, 56 men), Malaysia (government servants)	Gender and COVID-19-related stressors sig. moderated the relationship between lifestyle changes due to COVID-19 and EE [*F* (12, 1,595) = 20.49, *p* < 0.001] Sig. interaction between COVID-19-related stressors and magnitude of change regarding EE for women [*B* = 0.005, *F* (1, 1,595) = 5.29, *p* = 0.021] but not for men [*B* = −0.001, *F* (1, 1,595) = 0.48, *p* = 0.487]
Jacob and Panwar (124)^a^	EBPQ	***N* = 120** (60 women, 60 men), India	Women: M = 25.60 ± 5.31, men: M = 24.05 ± 5.08 ➔ no sig. gender difference (*p* = 0.10)
Karakose et al. (94)^a^	DEBQ	***N* = 266** (35 women, 231 men), Türkiye (school administrators)	Women: M = 2.07 ± 1.14, men: M = 1.70 ± 0.87 ➔ sig. gender difference (*p* = 0.024)
Liboredo et al. (91)	TFEQ-R21	***N* = 1,368** (1,094 women, 269 men, 5 “not specified”) Brazil	EE score <61.1: 757 (77.2%) women vs. 224 (22.8%) men EE score ≥61.1: 337 (88.2%) women vs. 45 (11.8%) men ➔ sig. association between gender and EE category (*p* < 0.01)
Lo Moro et al. (96)	EOQ	***N* = 1,865** (1,305 women, 560 men), Italy	Women had higher odds of emotional overeating compared to men [OR = 1.61; 95% CI (1.26; 2.05); *p* < 0.001]
Modrzejewska et al. (93)	EOQ	***N* = 868** (773 women, 95 men), Poland	Sig. correlation between gender and emotional overeating (*r* = 0.08, *p* < 0.05) Log. regression: gender was a sig. predictor of emotional overeating while controlling for BMI, visual- and attitude-related as well as body- and health-related predictors (*p* = 0.010); when emotion-related predictors were additionally controlled, gender was no longer sig.
Nashwan et al. (125)^a^	EEQ	***N* = 200** (81 women, 119 men), Qatar (nurses)	Women had significantly higher odds of higher EEQ categories compared to men [OR = 2.70; 95% CI (1.39; 5.26); *p* = 0.003]
Oliveira et al. (85)	TFEQ-R21	***N* = 1,363** (1,094 women, 269 men), Brazil	Only in men, the EE score was a sig. predictor of food cravings [OR = 1.04; 95% CI: (1.02; 1.05); *p* < 0.001]
Özcan and Yeşilkaya (82)^a^	EEQ	***N* = 578** (422 women, 156 men), Türkiye	Pre-pandemic: Women: M = 7.81 ± 4.05, men: M = 7.08 ± 2.91 ➔ no sig. gender difference (*p* = 0.091) Women: 88.2% low EE, 9.7% EE, 2.1% high EE, men: 96.2% low EE, 2.6% EE, 1.2% high EE ➔ sig. gender difference (*p* = 0.013) During the pandemic: Women: M = 20.17 ± 5.18, men: M = 18.31 ± 5.12 ➔ sig. gender difference (*p* < 0.001) EEQ score increased sig. in women and men (*p* < 0.001) Women: 3.3% low EE, 61.4% EE, 35.3% high EE, men: 8.3% low EE, 67.3% EE, 24.4% high EE ➔ sig. gender difference (*p* = 0.004)
Rahim et al. (99)	TFEQ-18	***N* = 3,738** (1,824 women, 1,914 men), Iraq	756 (41.5%) women vs. 729 (38.1%) men reported EE ➔ no sig. association between gender and EE category (*p* = 0.102)
Renzo et al. (102)	YFAS	***N* = 602** (480 women, 120 men, 2 “not specified” gender), Italy	Sig. more women reported EE than men (“use of food to respond to anxious feelings,” “need to increase food intake to feel better,” “anxious feelings due to current eating habits”) ➔ sig. gender difference (*p* < 0.001, respectively)
Ricalde-Castillo et al. (97)	EEQ	***N* = 500** (213 women, 287 men), Peru (nurses)	Women: M = 9.49 ± 4.76, men: M = 9.14 ± 4.53 ➔ no sig. gender difference (*p* = 0.400)
Serin and Koç (100)	DEBQ	***N* = 1,064** (624 women, 440 men), Türkiye (university students)	Women: median = 32.50 (IQR: 22.00, 40.00), men: median = 27.00 (IQR: 19.00, 36.00) ➔ sig. gender difference (*p* < 0.05)
Sumalla-Cano et al. (101)^a^	EEQ	***N* = 168** (112 women, 56 men), Spain (university population)	Non-/low emotional eater: 64.2% women vs. 89.2% men Emotional/very emotional eater: 35.7% women vs. 10.7% men ➔ sig. association between gender and EE category (*p* < 0.01)
Tazeoglu et al. (81)^a^	DEBQ	Pre-Quarantine: ***N* = 746** (397 women, 349 men) Post Quarantine: ***N* = 386** (203 women, 183 men), Türkiye (university students)	Pre-quarantine: Women: M = 2.51 ± 0.97, men: M = 2.06 ± 0.98 Post-quarantine: Women: M = 2.96 ± 0.84 ➔ sig. increase (*p* < 0.01), men: M = 2.52 ± 0.89 ➔ sig. increase (*p* < 0.01) EE score was sig. higher in women than in men post- and pre-quarantine, respectively, ➔ sig. gender difference (*p* < 0.001)
Usubini et al. (86)	DEBQ	***N* = 437** (224 women, 213 men), Italy (young adults)	Women: M = 2.38 ± 0.94, men: M = 1.74 ± 0.71 ➔ sig. difference (*p* < 0.001)
Vila-Marti et al. (79)	TFEQ-18	***N* = 9,849** (7,889 women, 1,960 men), 21 Spanish-speaking countries	EE < 9 points: 85.0% women vs. 15.0% men EE 9–10 points: 79.1% women vs. 20.9% men (prevalence ratio = 1.22) ➔ sig. gender difference (*p* < 0.001) EE > 10 points: 72.8% women vs. 27.2% men (prevalence ratio 1.11) ➔ sig. gender difference (*p* < 0.001)
Wu et al. (103)^a^	DEBQ	***N* = 1,135** (580 women, 555 men), China (university students)	Women: M = 26.3 ± 9.8, men: M = 22.5 ± 9.4 ➔ sig. gender difference (*p* < 0.01) Gender was a sig. predictor of EE (*β* = 0.16, *p* < 0.01)
Yoldas Ilktac et al. (88)	EES	***N* = 495** (342 women, 153 men), Türkiye	Sig. correlation between gender and EES score (*r* = 0.183; *p* < 0.01) Gender was a sig. predictor of EE (*β* = −0.169, *p* < 0.001)

EE, emotional eating; OR, odds-ratio; CI, confidence interval; IQR, interquartile range; EEQ, Emotional Eater Questionnaire (73); EES, Emotional Eating Scale (8); TFEQ, Three-Factor Eating Questionnaire, R18 (74), R21 (75); DEBQ, Dutch Eating Behavior Questionnaire (77); EBPQ, Eating Behavior Pattern Questionnaire (76); EOQ, Emotional Overeating Questionnaire (78); YFAS, Yale Food Addiction Scale (80).

a Studies included in meta-analysis. The bold values indicate the sample size (*N*) for each study. This was done to highlight the relevance of sample size for interpreting the results.

The most frequently used tools for assessing EE included the EEQ (73); *n* = 7, the TFEQ in its 18-item (TFEQ-R18) (74) or 21-item version (TEFQ-R21) (75); *n* = 6, and the DEBQ (77); *n* = 6. The EES (8) was used in four studies, while self-developed questions were employed in three. Two studies used the EOQ-5 (78), and one study each used the EBPQ (76), and the Yale Food Addiction Scale (YFAS) (80).

Among the studies, one was longitudinal (81), while the remaining 29 employed cross-sectional designs. Nonetheless, one cross-sectional study (82) included retrospective (pre-pandemic) and prospective (i.e., during the pandemic) assessments of EE. Study samples were primarily drawn from the general population (*n* = 17), with no specific age or profession criteria. Additional groups included university populations (*n* = 6), young adults (*n* = 2), nurses (*n* = 2), workers from the National Research Council (*n* = 1), government servants (*n* = 1), and school administrators (*n* = 1).

#### 3.2.1 Gender differences

Significant gender differences in EE behaviors were consistently observed across studies, with women typically reporting higher EE scores than men.

Multiple studies using the EEQ observed significant gender differences, with women scoring higher than men. For example, in Turkey, Özcan and Yeşilkaya (82) reported that women exhibited significantly higher EEQ scores during the COVID-19 pandemic, although no such difference was observed pre-pandemic, reflecting an increase in EE for both genders, with more pronounced changes among women. Similarly, in Ecuador (83), a significant association between gender and EE was found, with women again scoring higher than men. In Malaysia, Hamzaid et al. (84) demonstrated that gender and pandemic-related stressors significantly moderated the relationship between lifestyle changes and EE, particularly among women.

Studies employing the TFEQ also identified substantial gender differences in EE scores. In large multinational studies (36, 79), women consistently had higher EE scores and were more likely to report EE behaviors than men. At the same time, in Brazil, Oliveira et al. (85) found that EE scores significantly predicted food cravings in men, but not in women, highlighting potential gender differences in EE-related sequelae.

The DEBQ also revealed gender disparities in EE. For example, Usubini et al. (86) found that Italian women scored significantly higher than men. Similarly, in Israel (65), higher emotional eating in women compared to men was observed. Tazeoglu et al. (81) found that EE scores increased significantly post-quarantine for both genders in Turkey, with women consistently scoring higher.

Findings from studies using the EES showed more heterogeneous results concerning gender differences. In Turkey, Göbel et al. (87) and Yoldas Ilktac et al. (88) reported significantly higher EES scores in women and a stronger association between gender and EE tendencies. Additionally, Eşer Durmaz et al. (89) found that Turkish women who spent more time on social media exhibited higher EES scores, while no significant differences were noted for men. Conversely, Carpio-Arias et al. (90) found no significant relationship between gender and EE scores in Ecuador.

Studies utilizing either self-developed questions, the EBPQ, or the YFAS also supported the trend of higher EE prevalence in women. For instance, in a large Norwegian study with self-developed questions (64), women were nearly twice as likely as men to report EE, with gender serving as a significant predictor of EE.

Overall, across various measurement tools, women demonstrated higher EE tendencies than men, with gender frequently acting as a significant predictor of EE. A summary of gender differences in EE is presented in Table 3.

#### 3.2.2 Associations between emotional eating and contributing factors during the COVID-19 pandemic

This section examines the associations between EE and various contributing factors during the COVID-19 pandemic, without emphasizing gender-specific outcomes.

Several studies found strong associations between changes in food intake during the pandemic and EE. For example, Liboredo et al. (91) identified increased food intake as a significant predictor of EE, with an odds ratio of 3.6 [95% CI (1.8; 3.7)], indicating a substantial rise in EE linked to higher food consumption. Similarly, De Diego et al. (92) reported that greater consumption of savory snacks, bakery products, and sweet snacks was significantly associated with higher EE behaviors (*p* < 0.001, respectively), highlighting the impact of altered dietary habits on EE during the pandemic.

COVID-19-related stress also emerged as a significant factor influencing EE. Hadar-Shoval et al. (65) found a notable correlation between pandemic-related stressors and EE (*r* = 0.24; *p* < 0.001). Participants who experienced positive changes in alcohol consumption, sleep quality, or smoking showed significantly less EE than those with negative changes, though no significant association was found with changes in physical activity. Similarly, Modrzejewska et al. (93) identified pandemic-related stress as a predictor of emotional overeating, even when controlling for variables such as gender, BMI, and affect regulation. These findings underscore the role of pandemic-induced stress in EE behaviors.

Fear of COVID-19 was another factor linked to EE. Karakose et al. (94) demonstrated a significant association between fear of the virus and EE (*r* = 0.35; *p* < 0.01), with fear of COVID-19 serving as a significant predictor of EE. Similarly, Yoldas Ilktac et al. (88) found an association between fear of COVID-19 and EE (*r* = 0.09; *p* = 0.046). However, fear did not remain a significant predictor after controlling for variables such as sleep quality, BMI, age, and gender.

Additional pandemic-specific factors included quarantine duration and lifestyle changes. Özcan and Yeşilkaya (82) found a significant association between the length of quarantine and EE scores (*r* = 0.61; *p* = 0.025), suggesting that prolonged quarantine correlated with increased EE. Vila-Marti et al. (79) reported that lower sleep quality, reduced physical activity, higher screen time, and changes in the relationship with food were negatively linked with medium and high EE scores (*p* < 0.05, respectively). Among these, changes in sleep quality were key predictors of high EE scores, emphasizing the critical role of sleep in managing EE.

Finally, Ederer et al. (95) explored EE in response to loneliness or stress, finding a significant association between EE and weight changes during the pandemic. Those who frequently engaged in EE due to loneliness or stress had increased odds of both weight loss and weight gain (odds-ratios ranging from 1.62 to 4.61), suggesting a strong link between EE behaviors and weight fluctuations during this period.

### 3.3 Meta-analytic findings on emotional eating across genders

The meta-analysis included 16 studies (see Table 3) on EE, with a combined sample size of 53,381 participants, comprising 34,137 women and 19,244 men. The initial random-effects model (see Supplementary Figure 1) revealed a moderate overall effect size [Cohen’s *d* = 0.34, 95% CI (0.21, 0.48), *p* < 0.001], suggesting that EE significantly differed by gender across studies. However, this initial model exhibited a high level of heterogeneity (*I*^2^ = 96.99%), indicating substantial variability between the included studies. The Q-test for heterogeneity was also significant [*Q* (15) = 700.30, *p* < 0.001], further highlighting unexplained variability across studies (see Supplementary Figure 1).

To better understand and address the observed heterogeneity, a leave-one-out sensitivity analysis was conducted. In this stepwise approach, the study by Vila-Marti et al. (79) was excluded first, slightly reducing heterogeneity to *I*^2^ = 90.32%, with a comparable effect size estimate [Cohen’s *d* = 0.39, 95% CI (0.30, 0.48), *p* < 0.001]. As additional studies with a strong influence on heterogeneity were removed—specifically, Usubini et al. (86), Lo Moro et al. (96), and Ricalde-Castillo et al. (97)—the heterogeneity dropped substantially, reaching *I*^2^ = 41.87% with 12 remaining studies. At this point, heterogeneity was no longer statistically significant (*p* = 0.078). The effect size estimate at this stage remained significant [Cohen’s *d* = 0.38, 95% CI (0.33, 0.42), *p* < 0.001], indicating that the effect of gender on EE is robust across these relatively homogeneous studies.

**Figure 2:**
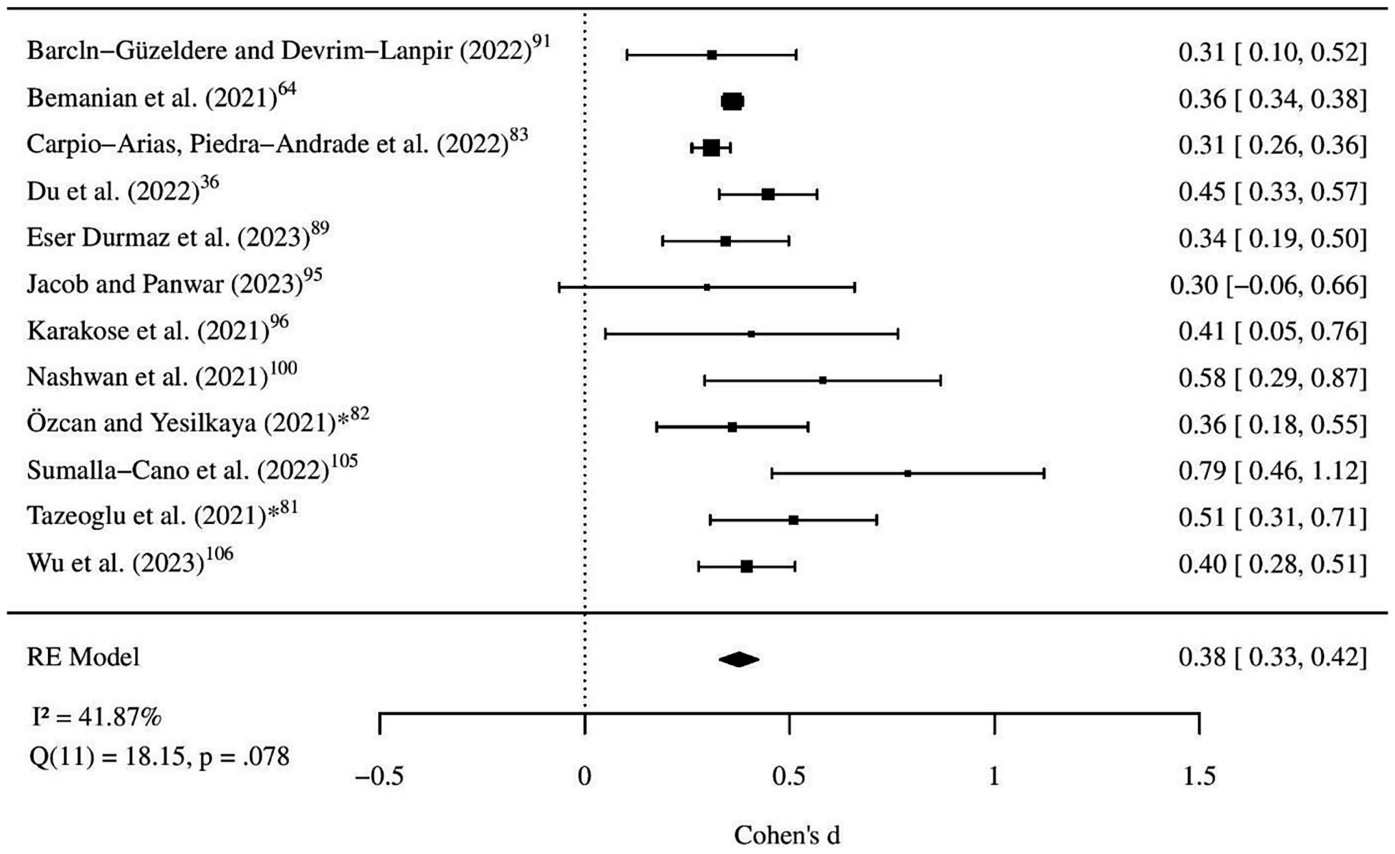
Forest plot of final random-effects model. All studies represent data collected during the COVID-19 pandemic. ^*^Indicates the second measurement point for Özcan and Yeşilkaya (82) and for Tazeoglu et al. (81).

The final exclusion involved omitting Carpio-Arias et al. (83), leaving a subset of 11 studies, which further reduced heterogeneity to *I*^2^ = 15.60%, while maintaining a stable effect size estimate [Cohen’s *d* = 0.39, 95% CI (0.35, 0.43), *p* < 0.001]. However, given the stability of the effect size and the lack of statistically significant heterogeneity (*p* = 0.078) with 12 studies, further reductions were deemed unnecessary. We selected the 12-study subset as the final analysis group (see Figure 2) to balance statistical homogeneity with the inclusion of a broader evidence base. This subset reflects a relatively homogeneous group of studies with consistent effect sizes, underscoring the reliability of the gender difference in EE across diverse study contexts and populations.

Subgroup analyses explored potential moderators to further explain variability in effect size across studies. When examining region as a moderator, this model accounted for 47.38% of the between-study heterogeneity. Notably, the “cross-regional Spanish-speaking” subgroup, represented solely by Vila-Marti et al. (79), showed a significant negative effect [estimate = −0.73, 95% CI (−1.26, −0.20), *p* = 0.007], indicating a reversal of the typical gender effect on EE, with men reporting higher levels than women. Importantly, other regional subgroups, such as studies from Spain categorized under “Europe” or studies from Latin America, did not yield significant effects when examined separately.

Analyses by sample type showed that samples consisting of young adults and university students had a significantly higher association with EE [estimate = 0.27, 95% CI (0.03, 0.51), *p* = 0.028] compared to the general population and professionals, suggesting that this demographic may be more susceptible to EE behaviors. Instrument type was also examined but did not significantly influence effect size, indicating that between-study variability was not strongly dependent on the measurement approach [QM (6) = 4.78, *p* = 0.573]. Full subgroup estimates (*k*, Cohen’s *d*, 95% CIs, *p*-values, *R*^2^/*I*^2^) are reported in Supplementary Table S1.

To investigate the combined effects of moderators on EE, a mixed-effects meta-regression was conducted. This model, which included both region and sample type as moderators, significantly reduced residual heterogeneity, explaining 71.10% of the variability. Within this model, the study from the Spanish-speaking subgroup Vila-Marti et al. (79) exhibited a significantly negative effect size (estimate = −0.49, *p* = 0.029), whereas young adults and university students showed a significantly higher effect size (estimate = 0.24, *p* = 0.016), consistent with findings from the subgroup analyses. The residual heterogeneity in this combined model remained modest (*I*^2^ = 77.61%, *p* < 0.001), indicating that while a substantial portion of variability is explained by these moderators, additional unmeasured factors may still contribute to differences across studies.

**Figure 3:**
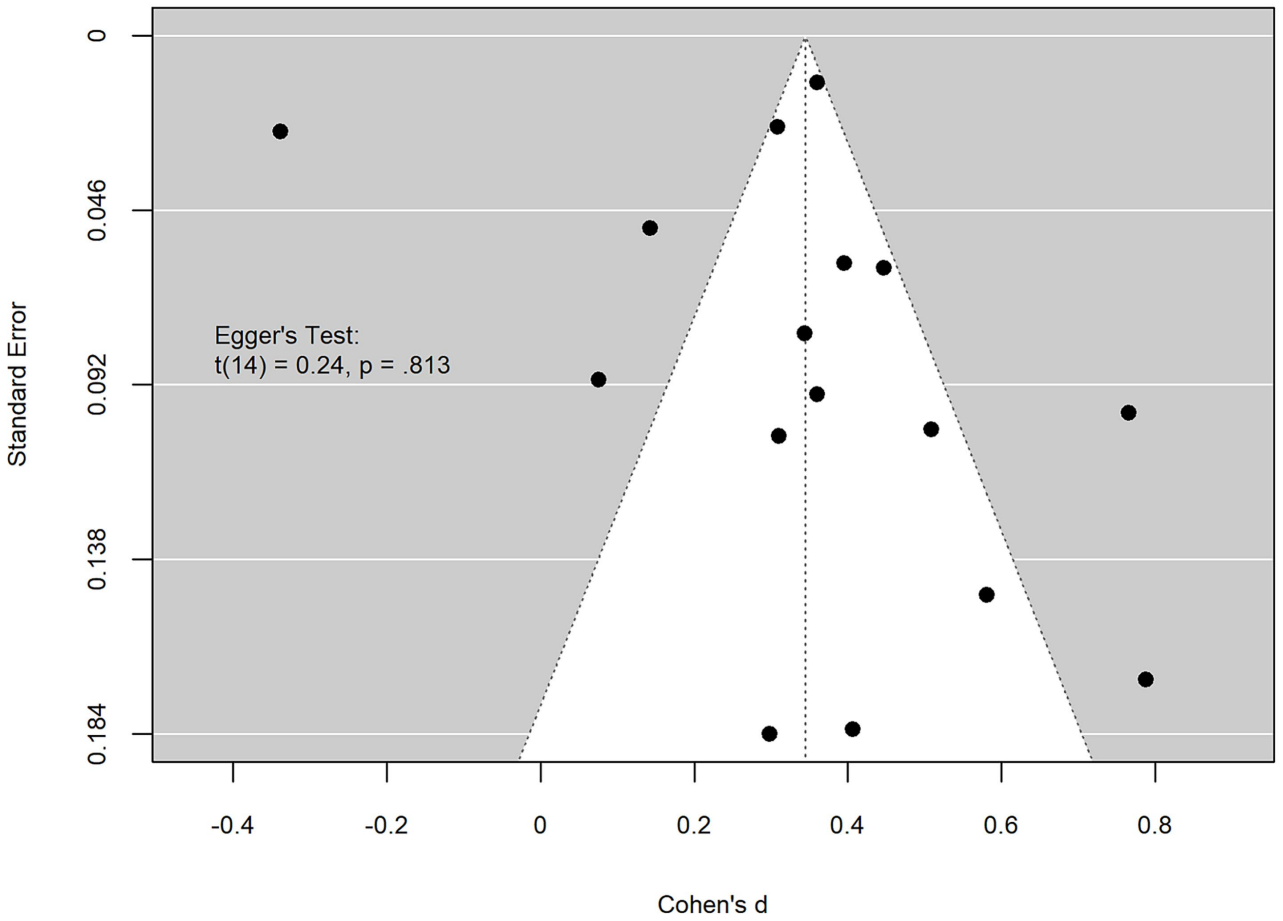
Funnel plot for potential publication bias.

Lastly, an examination of a potential publication bias was conducted. A funnel plot analysis, along with Egger’s test for funnel plot asymmetry [*t* (14) = 0.241, *p* = 0.813], suggested no evidence of publication bias in the included studies, indicating that the observed results are unlikely to be influenced by selective publication practices (see Figure 3).

### 3.4 Quality assessment

The quality assessment of the included studies revealed variations in adherence to established evaluation criteria. Most studies met key quality standards, including clear definitions of the study population, detailed descriptions of participants and settings, identification of confounding factors, valid and reliable outcome measurements, and the use of appropriate statistical methods.

However, specific shortcomings were identified in several studies. Notably, some studies lacked explicit definitions of sample inclusion criteria, including those by Bemanian et al. (64), Özcan and Yeşilkaya (82), Elmacloǧlu et al. (98), Karakose et al. (94), Lo Moro et al. (96), Modrzejewska et al. (93), Rahim et al. (99), Serin and Koç (100), and Sumalla-Cano et al. (101). Additionally, deficiencies in the identification and management of confounding variables were common. On the other hand, the following studies fulfilled both quality criteria: Hadar-Shoval et al. (65), Eşer Durmaz et al. (89), Carpio-Arias et al. (90), Ederer et al. (95), Liboredo et al. (91), Modrzejewska et al. (93), Renzo et al. (102), Ricalde-Castillo et al. (97), and Wu et al. (103).

Concerns regarding the validity and reliability of outcome measurements were limited, with only De Diego et al. (92) showing notable issues in this area. A detailed summary of the quality assessment for each study is presented in Table 4.

**Table 4:** Quality assessment.

References	Were the criteria for inclusion in the sample clearly defined?	Were the study subjects and the setting described in detail?	Were confounding factors identified?	Were strategies to deal with confounding factors stated?	Were the outcomes measured in a valid and reliable way?	Was appropriate statistical analysis used?
Barcln-Güzeldere and Devrim Lanpir (123)	Yes	Yes	No	No	Yes	Yes
Bemanian et al. (64)	No	Yes	Yes	?	Yes	Yes
Carpio-Arias et al. (83)	Yes	Yes	No	No	Yes	Yes
Carpio-Arias et al. (90)	Yes	Yes	Yes	Yes	Yes	Yes
De Diego et al. (92)	Yes	Yes	Yes	?	No	Yes
Du et al. (36)	Yes	Yes	?	?	Yes	Yes
Ederer et al. (95)	Yes	Yes	Yes	Yes	Yes	Yes
Elmacloǧlu et al. (98)	No	Yes	No	No	Yes	Yes
Eşer Durmaz et al. (89)	Yes	Yes	Yes	Yes	Yes	Yes
Göbel et al. (87)	Yes	No	?	No	Yes	Yes
Hadar-Shoval et al. (65)	Yes	Yes	Yes	Yes	Yes	Yes
Hamzaid et al. (84)	Yes	Yes	No	No	Yes	Yes
Jacob and Panwar (124)	Yes	Yes	Yes	?	Yes	Yes
Karakose et al. (94)	No	Yes	No	No	Yes	No
Liboredo et al. (91)	Yes	Yes	Yes	Yes	Yes	Yes
Lo Moro et al. (96)	No	Yes	No	No	Yes	Yes
Modrzejewska et al. (93)	No	Yes	Yes	Yes	Yes	Yes
Nashwan et al. (125)	Yes	Yes	?	?	Yes	Yes
Oliveira et al. (85)	Yes	Yes	No	No	Yes	Yes
Özcan and Yeşilkaya (82)	No	?	No	No	Yes	Yes
Rahim et al. (99)	No	Yes	No	No	Yes	Yes
Renzo et al. (102)	Yes	Yes	Yes	Yes	Yes	Yes
Ricalde-Castillo et al. (97)	Yes	Yes	Yes	Yes	Yes	Yes
Serin and Koç (100)	No	?	No	No	Yes	?
Sumalla-Cano et al. (101)	No	Yes	Yes	No	Yes	Yes
Tazeoglu et al. (81)	Yes	Yes	No	No	Yes	Yes
Usubini et al. (86)	Yes	Yes	Yes	?	Yes	Yes
Vila-Marti et al. (79)	Yes	Yes	?	No	Yes	?
Wu et al. (103)	Yes	Yes	Yes	Yes	Yes	Yes
Yoldas Ilktac et al. (88)	Yes	Yes	Yes	?	Yes	Yes

? = unclear. As the exposure in our study is the COVID-19 pandemic, this aspect (i.e., “Was the exposure measured in a valid and reliable way?”) was removed from the quality assessment template. In a similar vein, as the population was the general population and we excluded clinical groups, the question regarding the assessment of the condition (“Were objective, standard criteria used for measurement of the condition?”) was removed.

## 4 Discussion

### 4.1 General findings

This systematic review and meta-analysis aimed to provide a comprehensive evaluation of gender differences in EE during the COVID-19 pandemic within an adult general population. Our study provides robust evidence for significant gender differences in EE during the COVID-19 pandemic. Across the large majority of the included studies, women reported higher levels of EE than men, a trend intensified by pandemic-specific stressors such as increased anxiety, fear, loneliness, and disrupted routines (65, 82, 95). While most studies supported the evidence of gender disparities, exceptions were noted. For instance, Carpio-Arias et al. (90) found no significant gender differences in the general population, and Elmacloǧlu et al. (98) observed no changes in EE due to social isolation. However, the overall trend indicates that women were disproportionately affected by EE during the pandemic.

Several studies, e.g., Hadar-Shoval et al. (65), Vila-Marti et al. (79), Özcan and Yeşilkaya (82), Hamzaid et al. (84), Ederer et al. (95), Karakose et al. (94), and Modrzejewska et al. (93), reported that gender and pandemic-related stressors such as increased anxiety and fear, sleep disturbances and loneliness, were significant moderators of EE behavior. Women’s tendency to rely on EE as a coping mechanism is consistent with previous research highlighting their greater predisposition toward emotion-focused coping strategies when faced with stressors (37).

The regional aspect of these studies further highlights the complexity of gender differences in EE. One notable finding was from Vila-Marti et al. (79), which uniquely identified a reversal of the typical gender effect on EE within Spanish-speaking populations spanning Spain and Latin America. In this study, men reported higher mean levels of EE than women; however, women were still more likely to be categorized in the high EE group. This result underscores the potential influence of cultural factors, such as differences in emotional expression or dietary habits, on EE behaviors. Importantly, the other regional subgroups, such as Europe and Latin America examined separately, did not yield significant effects, suggesting that geographic (i.e., cultural) region may not uniformly influence gender differences in EE. Nonetheless, our study encompasses a diverse range of geographical contexts, including the Middle East, East Asia, South Asia, and cross-regional datasets, providing a broad perspective on EE behaviors. This geographical diversity strengthens the generalizability of the findings while highlighting areas for further exploration of cultural moderators of EE.

In addition, Oliveira et al. (85) identified EE as a significant predictor of food cravings in men but not in women in a Brazilian sample, highlighting further the nuanced ways in which EE may manifest across genders. This finding may reflect the distinction between EE and food cravings, where EE involves eating in response to emotional triggers like stress or anxiety, while food cravings are characterized by intense, specific desires for particular foods, often driven by physiological or sensory cues. Therefore, the results reported by Oliveira et al. (85) suggest that men’s EE might be more closely linked to food cravings, potentially due to gender differences in how emotional and physiological responses to food are processed.

The observed gender differences in EE during the COVID-19 pandemic can be partly explained by divergent stress-coping strategies. Women are more likely to engage in avoidant, passive, and emotion-focused coping mechanisms, such as self-blame and behavioral disengagement (104, 105), which can increase anxiety (58). Consequently, women are more inclined to engage in EE as a way to temporarily alleviate negative emotions such as stress, anger, anxiety, or sadness (21, 37). While this behavior may offer momentary relief, it fails to address the root causes of these stressors. Pandemic-related stressors, such as social isolation, increased caregiving responsibilities, and economic uncertainty (106), likely intensified this reliance on EE. In contrast, men tend to use more problem-focused strategies, such as active planning, seeking instrumental support, and directly confronting challenges, which are generally linked to better mental health outcomes (104, 105). However, when men do engage in EE, it tends to manifest as reward-seeking behavior, where consuming calorie-dense foods satisfies cravings and provides immediate satisfaction (85). This is consistent with physiological evidence suggesting gender differences in appetite responses to negative emotions, with women reporting increased appetite and men experiencing appetite suppression (107, 108), which may partly explain their lower EE scores.

It is challenging to determine the extent to which observed changes were directly caused by the pandemic. Only a limited number of studies were able to assess changes in gender differences in EE over time. For instance, Tazeoglu et al. (81) observed that both men and women significantly increased their EE during the quarantine period, with women consistently scoring higher than men both before and after the quarantine. Notably, this study did not find evidence of a significant exacerbation of the gender gap in EE scores during this specific period. In contrast, Özcan and Yeşilkaya (82), by comparing EE behaviors before and during the pandemic, identified a trend toward gender-specific changes. Their findings suggest that EE increased for both men and women, with a more pronounced rise in women, indicating that the pandemic may have intensified pre-existing gender-specific coping tendencies. Elmacloǧlu et al. (98) found no significant gender differences in changes in EE due to social isolation, while Hamzaid et al. (84) demonstrated a significant interaction between COVID-19-related stressors and the extent of change in EE for women (*p* = 0.021) but not for men (*p* = 0.487).

Preexisting mental health conditions likely intensified EE among women during the pandemic. Reflecting the overall higher prevalence of depression and anxiety in women (109, 110), research indicates that women experienced significantly higher levels of depression, anxiety, and stress during the first wave of the pandemic (111). This increase was partly due to heightened concerns for family well-being and increased caregiving responsibilities, which disproportionately impacted women (112). Additionally, restricted access to healthcare services during the pandemic contributed to increased anxiety and a general worsening of mental health conditions. This combination of heightened psychological stress and limited support may have led women with preexisting mental health conditions to engage in EE more frequently as a coping mechanism.

The meta-analytic findings— including 16 studies—revealed that the gender differences (with a stable effect size of Cohen’s *d* = 0.4) in EE were more pronounced in studies focusing on young adults and students compared to those investigating the general population and professionals. Several interconnected factors may explain this heightened effect among younger adults. Developmentally, young adulthood is a critical period marked by heightened emotional reactivity and limited impulse control, which may predispose individuals to maladaptive coping mechanisms such as EE (113, 114). For young women in particular, societal pressures related to body image and appearance, combined with greater tendencies toward emotion-focused coping strategies, may amplify their susceptibility to EE compared to young men (62, 63). Moreover, pandemic-specific stressors, such as disruptions to education, early career paths, and social connections, disproportionately impacted younger adults (115, 116). These combined biological, psychological, and sociocultural factors likely contributed to the observed intensified gender differences in EE within younger adult populations, underscoring the need for targeted interventions tailored to this demographic.

Based on the results of this study, important implications arise for public health strategies designed to mitigate the long-term health risks associated with EE. The elevated gender disparities observed in young adult samples emphasize the urgency of developing tailored, gender-specific interventions aimed at improving emotion regulation and stress management. Women’s heightened reliance on EE as a coping mechanism highlights the need for targeted interventions during crises, addressing both immediate emotional needs and the underlying stressors. Given the established link between EE and obesity, as well as the greater burden of obesity-related health conditions on women (117), such strategies are crucial for reducing health disparities and promoting equitable health outcomes (118).

Young adults and university students, identified as particularly vulnerable to EE, require special consideration in public health initiatives. The greater gender disparities observed in this demographic suggest that age-related developmental factors, such as higher emotional reactivity and less effective impulse control, may exacerbate the tendency toward EE (119), particularly in young adults (120). These findings point to the necessity of resilience-building programs that integrate gender-sensitive approaches, providing education on stress management, emotion regulation, and healthy coping strategies. Establishing support networks and increasing access to mental health services tailored for this age group could mitigate the negative impacts of EE, reduce gender disparities, and foster healthier long-term behaviors.

Our findings also point to the importance of tailoring interventions to gender-specific needs. For women, who more frequently report EE as a coping strategy, interventions may focus on strengthening adaptive coping mechanisms and stress regulation. For men, however, the lower reported levels of EE might partly reflect difficulties in perceiving, labeling, or acknowledging emotional states. This suggests that interventions for men may benefit from emphasizing emotional literacy and recognition, ensuring that maladaptive eating patterns are not overlooked simply because they are not self-identified as “emotional eating.” By addressing both coping and awareness, gender-sensitive programs can more effectively reduce EE-related health risks and promote equitable outcomes.

### 4.2 Limitations and future directions

Several limitations should be noted when interpreting the findings of this systematic review and meta-analysis. First, most of the included studies were cross-sectional, which limits the ability to establish causal relationships between pandemic conditions and changes in EE. Cross-sectional designs capture data at a single point in time and cannot account for changes in eating behaviors compared to pre-pandemic baselines, potentially introducing bias (121, 122). Longitudinal research during times of crises is essential to elucidate the causal and gender-specific pathways linking pandemic-related stressors to psychopathology, e.g., EE behaviors. In addition, we did not systematically search the grey literature (e.g., dissertations, reports, preprints). This may have excluded relevant non-indexed studies and increased the risk of publication bias. However, we formally assessed publication bias (funnel plot and Egger’s test), and the results did not indicate substantial bias, which mitigates this concern. Another limitation is the potential selection bias within the studies included in the meta-analysis, as many focused on specific populations, such as students, certain geographic regions, or professional groups, which may limit their representativeness for the general population. This limits the generalizability of the findings to broader or more diverse groups. Furthermore, the over-representation of certain demographic groups, such as younger adults, may have influenced the results, as EE is often more prevalent in these populations. To address this limitation, we conducted subgroup analyses to explore potential variations across different demographic groups and regions.

The quality assessment identified variations in adherence to the defined evaluation criteria. While most studies fulfilled key quality standards, notable shortcomings were observed. The most prevalent issues included unclear inclusion criteria and inadequate consideration of confounding factors, which may have implications for the robustness of the findings.

Self-report measures used in the included studies, such as the EEQ, TFEQ, DEBQ, and EES may also be subject to bias. Participants may underreport or overreport their behaviors due to social desirability or recall bias, particularly in the context of sensitive topics such as EE. This may affect the accuracy of the reported gender differences and undermine the reliability of the conclusions. The variation in measurement tools across studies is another limitation. Different instruments assess EE in slightly different ways, which may contribute to variability in the results. While tools like the EEQ, TFEQ, DEBQ, and EES are widely used, inconsistencies in how EE is conceptualized and measured could affect the comparability of the findings across studies. To mitigate this limitation, again, we conducted subgroup analyses to examine the potential impact of different measurement tools on the results.

Moreover, pandemic-related stressors, such as social isolation and caregiving responsibilities, were found to exacerbate EE in women (84, 95), however, the role of other psychological and social factors, such as pre-existing mental health conditions and socioeconomic status, has not been fully explored. Future research should consider these factors, as they may further contribute to gender differences in EE during stressful events like a pandemic.

Finally, although some included studies mentioned categories beyond the binary gender classification (e.g., “other” or “not specified” gender), none provided detailed analyses for these groups. As a result, our meta-analysis could only address gender differences between women and men. This limitation highlights the urgent need for future studies to systematically include and analyze data from individuals with diverse gender identities to better capture the full spectrum of EE behaviors.

## 5 Conclusion

This review provides valuable insights into gender differences in EE in the general population during the COVID-19 pandemic, combining a robust narrative synthesis with a meta-analysis that spans diverse populations and geographical regions. By examining cross-country data and emphasizing gender-specific differences, this study contributes to a deeper understanding of how pandemic-related stressors have uniquely affected EE. The inclusion of studies utilizing a variety of methodologies further strengthens the reliability of the findings. Despite these contributions, several limitations highlight the need for further research, particularly longitudinal studies, to better understand causal pathways and gender-specific responses to stressors. Addressing these gaps in future research will be crucial to developing more effective, gender-sensitive approaches to manage EE and its associated health risks.

## Data Availability

The datasets generated and analyzed during the current study are available from the corresponding author on reasonable request. Access to some data may be restricted due to copyright or data protection regulations. Requests to access these datasets should be directed to christopher.zaiser@ruhr-uni-bochum.de.

## References

[1] Cucinotta D Vanelli M. Who declares COVID-19 a pandemic. Acta Biomed. (2020) 91:157–60. doi: 10.23750/abm.v91i1.9397, PMID: 32191675 PMC7569573

[2] Harder T Külper-Schiek W Reda S Treskova-Schwarzbach M Koch J Vygen-Bonnet S . Effectiveness of COVID-19 vaccines against SARS-CoV-2 infection with the Delta (B.1.617.2) variant: second interim results of a living systematic review and meta-analysis, 1 January to 25 August 2021. Euro Surveill. (2021) 26:2100920. doi: 10.2807/1560-7917.ES.2021.26.41.2100920 34651577 PMC8518304

[3] Caroppo E Mazza M Sannella A Marano G Avallone C Claro AE . Will nothing be the same again?: changes in lifestyle during COVID-19 pandemic and consequences on mental health. Int J Environ Res Public Health. (2021) 18:8433. doi: 10.3390/ijerph18168433, PMID: 34444180 PMC8393535

[4] Ashraf BN. Economic impact of government interventions during the COVID-19 pandemic: international evidence from financial markets. J Behav Exp Finance. (2020) 27:100371. doi: 10.1016/j.jbef.2020.100371, PMID: 32835011 PMC7323651

[5] Manchia M Gathier AW Yapici-Eser H Schmidt MV de Quervain D van Amelsvoort T . The impact of the prolonged COVID-19 pandemic on stress resilience and mental health: a critical review across waves. Eur Neuropsychopharmacol. (2022) 55:22–83. doi: 10.1016/j.euroneuro.2021.10.864, PMID: 34818601 PMC8554139

[6] Gao Y Bagheri N Furuya-Kanamori L. Has the COVID-19 pandemic lockdown worsened eating disorders symptoms among patients with eating disorders? A systematic review. J Public Health. (2022) 30:2743–52. doi: 10.1007/s10389-022-01704-4, PMID: 35369670 PMC8961480

[7] Ramalho SM Trovisqueira A de Lourdes M Gonçalves S Ribeiro I Vaz AR . The impact of COVID-19 lockdown on disordered eating behaviors: the mediation role of psychological distress. Eat Weight Disord. (2022) 27:179–88. doi: 10.1007/s40519-021-01128-1, PMID: 33713336 PMC7955211

[8] Arnow B Kenardy J Agras WS. The emotional eating scale: The development of a measure to assess coping with negative affect by eating. Int J Eat Disord. (1995) 18:79–90. doi: 10.1002/1098-108X(199507)18:1<79::AID-EAT2260180109>3.0.CO;2-V, PMID: 7670446

[9] Madalı B Alkan ŞB Örs ED Ayrancı M Taşkın H Kara HH. Emotional eating behaviors during the COVID-19 pandemic: a cross-sectional study. Clin Nutr ESPEN. (2021) 46:264–70. doi: 10.1016/j.clnesp.2021.09.745, PMID: 34857207 PMC8492000

[10] Varela C Andrés A Saldaña C. The behavioral pathway model to overweight and obesity: coping strategies, eating behaviors and body mass index. Eat Weight Disord. (2020) 25:1277–83. doi: 10.1007/s40519-019-00760-2, PMID: 31376111

[11] Chao A Grey M Whittemore R Reuning-Scherer J Grilo CM Sinha R. Examining the mediating roles of binge eating and emotional eating in the relationships between stress and metabolic abnormalities. J Behav Med. (2016) 39:320–32. doi: 10.1007/s10865-015-9699-1, PMID: 26686376 PMC5576015

[12] Wiedemann AA Ivezaj V Grilo CM. An examination of emotional and loss-of-control eating after sleeve gastrectomy surgery. Eat Behav. (2018) 31:48–52. doi: 10.1016/j.eatbeh.2018.07.008, PMID: 30118925 PMC6247803

[13] Crowley N Madan A Wedin S Correll JA Delustro LM Borckardt JJ . Food cravings among bariatric surgery candidates. Eat Weight Disord. (2014) 19:371–6. doi: 10.1007/s40519-013-0095-y, PMID: 24446333

[14] Camilleri GM Méjean C Kesse-Guyot E Andreeva VA Bellisle F Hercberg S . The associations between emotional eating and consumption of energy-dense snack foods are modified by sex and depressive symptomatology. J Nutr. (2014) 144:1264–73. doi: 10.3945/jn.114.193177, PMID: 24850627

[15] O’Connor DB Jones F Conner M Mcmillan B Ferguson E. Effects of daily hassles and eating style on eating behavior. Health Psychol. (2008) 27:S20–31. doi: 10.1037/0278-6133.27.1.S20 18248102

[16] Bullock AJ Goldbacher EM. Interoceptive awareness and emotional eating in college women: the role of appetite and emotional awareness. J Am Coll Heal. (2023) 71:2445–50. doi: 10.1080/07448481.2021.1970566, PMID: 34469250

[17] Jenkinson PM Taylor L Laws KR. Self-reported interoceptive deficits in eating disorders: a meta-analysis of studies using the eating disorder inventory. J Psychosom Res. (2018) 110:38–45. doi: 10.1016/j.jpsychores.2018.04.005, PMID: 29764604

[18] Brewer R Cook R Bird G. Alexithymia: a general deficit of interoception. R Soc Open Sci. (2016) 3:150664. doi: 10.1098/rsos.150664, PMID: 27853532 PMC5098957

[19] Heatherton TE Baumeister RF. Binge eating as escape from self-awareness. Psychol Bull. (1991) 110:8–108. doi: 10.1037/0033-2909.110.1.86 1891520

[20] Finch LE Tomiyama AJ. Stress-induced eating dampens physiological and behavioral stress responses In: Nutrition in the prevention and treatment of abdominal obesity. 2nd ed. Amsterdam: Elsevier Inc. (2014). 189–95.

[21] Klatzkin RR Nolan LJ Kissileff HR. Self-reported emotional eaters consume more food under stress if they experience heightened stress reactivity and emotional relief from stress upon eating. Physiol Behav. (2022) 243:113638. doi: 10.1016/j.physbeh.2021.113638, PMID: 34742909 PMC8717738

[22] Altheimer G Urry HL. Do emotions cause eating? The role of previous experiences and social context in emotional eating. Curr Dir Psychol Sci. (2019) 28:234–40. doi: 10.1177/0963721419837685

[23] Jackson A Anderson A Weybright E Lanigan J. Differing experiences of boredom during the pandemic and associations with dietary behaviors. J Nutr Educ Behav. (2021) 53:706–11. doi: 10.1016/j.jneb.2021.04.005, PMID: 34162515

[24] Schneider KL Appelhans BM Whited MC Oleski J Pagoto SL. Trait anxiety, but not trait anger, predisposes obese individuals to emotional eating. Appetite. (2010) 55:701–6. doi: 10.1016/j.appet.2010.10.006, PMID: 20959131 PMC3030203

[25] Dakanalis A Mentzelou M Papadopoulou SK Papandreou D Spanoudaki M Vasios GK . The association of emotional eating with overweight/obesity, depression, anxiety/stress, and dietary patterns: a review of the current clinical evidence. Nutrients. (2023) 15:1173. doi: 10.3390/nu15051173, PMID: 36904172 PMC10005347

[26] Tan CC Chow CM. Stress and emotional eating: the mediating role of eating dysregulation. Pers Individ Dif. (2014) 66:1–4. doi: 10.1016/j.paid.2014.02.033

[27] Miniati M Marzetti F Palagini L Marazziti D Orrù G Conversano C . Eating disorders spectrum during the COVID pandemic: a systematic review. Front Psychol. (2021) 12:663376. doi: 10.3389/fpsyg.2021.663376, PMID: 34658992 PMC8511307

[28] Muscogiuri G Barrea L Aprano S Framondi L di Matteo R Laudisio D . Sleep quality in obesity: does adherence to the Mediterranean diet matter? Nutrients. (2020) 12:1364. doi: 10.3390/nu12051364, PMID: 32397621 PMC7284844

[29] Rodríguez-Hidalgo AJ Pantaleón Y Dios I Falla D. Fear of COVID-19, stress, and anxiety in university undergraduate students: a predictive model for depression. Front Psychol. (2020) 11:591797. doi: 10.3389/fpsyg.2020.591797, PMID: 33224080 PMC7674167

[30] Moynihan AB van Tilburg WAP Igou ER Wisman A Donnelly AE Mulcaire JB. Eaten up by boredom: consuming food to escape awareness of the bored self. Front Psychol. (2015) 6:369. doi: 10.3389/fpsyg.2015.00369, PMID: 25883579 PMC4381486

[31] Yılmaz C Gökmen V. Neuroactive compounds in foods: occurrence, mechanism and potential health effects. Food Res Int. (2020) 128:108744. doi: 10.1016/j.foodres.2019.108744, PMID: 31955786

[32] Richardson C Patton M Phillips S Paslakis G. The impact of the COVID-19 pandemic on help-seeking behaviors in individuals suffering from eating disorders and their caregivers. Gen Hosp Psychiatry. (2020) 67:136–40. doi: 10.1016/j.genhosppsych.2020.10.006, PMID: 33129138 PMC10277602

[33] Richardson C Phillips S Paslakis G. One year in: The impact of the COVID-19 pandemic on help-seeking behaviors among youth experiencing eating disorders and their caregivers. Psychiatry Res. (2021) 306:114263. doi: 10.1016/j.psychres.2021.114263, PMID: 34758404 PMC8572344

[34] Devoe DJ Anderson A Bahji A Singh M Patten SB Soumbasis A . The prevalence of impulse control disorders and behavioral addictions in eating disorders: a systematic review and meta-analysis. Front Psychiatry. (2022) 12:724034. doi: 10.3389/fpsyt.2021.724034, PMID: 35069274 PMC8770943

[35] Laskowski NM Brandt G Reque CB Sabel L Pahlenkemper M Zaiser C . The collateral effects of the COVID-19 pandemic: a gender-specific systematic review of disordered eating behaviour in the general population. Eur Eat Disord Rev. (2024) 33:254–88. doi: 10.1002/erv.3141, PMID: 39436325 PMC11786942

[36] Du C Adjepong M Zan MCH Cho MJ Fenton JI Hsiao PY . Gender differences in the relationships between perceived stress, eating behaviors, sleep, dietary risk, and body mass index. Nutrients. (2022) 14:1045. doi: 10.3390/nu14051045, PMID: 36235535 PMC9570927

[37] Guerrero-Hreins E Stammers L Wong L Brown RM Sumithran P. A comparison of emotional triggers for eating in men and women with obesity. Nutrients. (2022) 14:4144. doi: 10.3390/nu14194144, PMID: 36235796 PMC9570591

[38] Larsen JK Van Strien T Eisinga R Engels RCME. Gender differences in the association between alexithymia and emotional eating in obese individuals. J Psychosom Res. (2006) 60:237–43. doi: 10.1016/j.jpsychores.2005.07.006, PMID: 16516654

[39] Thompson S. Gender and racial differences in emotional eating, food addiction symptoms, and body weight satisfaction among undergraduates. J Diabetes Obes. (2015) 2:1–6. doi: 10.15436/2376-0494.15.035

[40] Smith JM Serier KN Belon KE Sebastian RM Smith JE. Evaluation of the relationships between dietary restraint, emotional eating, and intuitive eating moderated by sex. Appetite. (2020) 155:104817. doi: 10.1016/j.appet.2020.104817, PMID: 32739329

[41] Rosenqvist E Kiviruusu O Berg N Konttinen H. Stress-induced eating and drinking and their associations with weight among women and men during 30-year follow-up. Psychol Health. (2023) 40:1–16. doi: 10.1080/08870446.2023.2192240, PMID: 36946279

[42] Braden A Musher-Eizenman D Watford T Emley E. Eating when depressed, anxious, bored, or happy: are emotional eating types associated with unique psychological and physical health correlates? Appetite. (2018) 125:410–7. doi: 10.1016/j.appet.2018.02.022, PMID: 29476800

[43] Wang L Shi C Zhang K Xu Q. The gender-specific association of EHD3 polymorphisms with major depressive disorder. Neurosci Lett. (2014) 567:11–4. doi: 10.1016/j.neulet.2014.02.055, PMID: 24607927

[44] Wong L Stammers L Churilov L Price S Ekinci E Sumithran P. Emotional eating in patients attending a specialist obesity treatment service. Appetite. (2020) 151:104708. doi: 10.1016/j.appet.2020.104708, PMID: 32283188

[45] Araujo DMR da Silva Santos GF Nardi AE. Binge eating disorder and depression: a systematic review. World J Biol Psychiatry. (2010) 11:199–207. doi: 10.3109/15622970802563171 20218783

[46] Brandt G Halbeisen G Braks K Huber TJ Paslakis G. Gender differences in individuals with obesity and binge eating disorder: a retrospective comparison of phenotypical features and treatment outcomes. Eur Eat Disord Rev. (2023) 31:413–24. doi: 10.1002/erv.2972, PMID: 36802088

[47] Fischer AH Kret ME Broekens J. Gender differences in emotion perception and self-reported emotional intelligence: a test of the emotion sensitivity hypothesis. PLoS One. (2018) 13:e0190712. doi: 10.1371/journal.pone.0190712, PMID: 29370198 PMC5784910

[48] Cholankeril R Xiang E Badr H. Gender differences in coping and psychological adaptation during the COVID-19 pandemic. Int J Environ Res Public Health. (2023) 20:993. doi: 10.3390/ijerph20020993, PMID: 36673747 PMC9859033

[49] Ling J Zahry NR. Relationships among perceived stress, emotional eating, and dietary intake in college students: eating self-regulation as a mediator. Appetite. (2021) 163:105215. doi: 10.1016/j.appet.2021.105215, PMID: 33774134

[50] Troisi A. Gender differences in vulnerability to social stress a Darwinian perspective. Physiol Behav. (2001) 73:443–9. doi: 10.1016/s0031-9384(01)00459-0 11438373

[51] Dedovic K Wadiwalla M Engert V Pruessner JC. The role of sex and gender socialization in stress reactivity. Dev Psychol. (2009) 45:45–55. doi: 10.1037/a0014433, PMID: 19209989

[52] Juster RP de Torre MB Kerr P Kheloui S Rossi M Bourdon O. Sex differences and gender diversity in stress responses and allostatic load among workers and LGBT people. Curr Psychiatry Rep. (2019) 21:110. doi: 10.1007/s11920-019-1104-2, PMID: 31630247

[53] Wang J Korczykowski M Rao H Fan Y Pluta J Gur RC . Gender difference in neural response to psychological stress. Soc Cogn Affect Neurosci. (2007) 2:227–39. doi: 10.1093/scan/nsm018, PMID: 17873968 PMC1974871

[54] Heck AL Handa RJ. Sex differences in the hypothalamic–pituitary–adrenal axis’ response to stress: an important role for gonadal hormones. Neuropsychopharmacology. (2019) 44:45–58. doi: 10.1038/s41386-018-0167-9, PMID: 30111811 PMC6235871

[55] Martinez-Muniz GA Wood SK. Sex differences in the inflammatory consequences of stress: implications for pharmacotherapy. J Pharmacol Exp Ther. (2020) 375:161–74. doi: 10.1124/JPET.120.266205, PMID: 32759370 PMC7569308

[56] Butler LD Nolen-Hoeksema S. Gender differences in responses to depressed mood in a college sample. Sex Roles. (1994) 30:331–46. doi: 10.1007/BF01420597

[57] Thomsen DK Mehlsen MY Viidik A Sommerlund B Zachariae R. Age and gender differences in negative affect—is there a role for emotion regulation? Pers Individ Dif. (2005) 38:1935–46. doi: 10.1016/j.paid.2004.12.001

[58] Kelly MM Tyrka AR Price LH Carpenter LL. Sex differences in the use of coping strategies: predictors of anxiety and depressive symptoms. Depress Anxiety. (2008) 25:839–46. doi: 10.1002/da.20341, PMID: 17603810 PMC4469465

[59] Cecchetto C Aiello M Gentili C Ionta S Osimo SA. Increased emotional eating during COVID-19 associated with lockdown, psychological and social distress. Appetite. (2021) 160:105122. doi: 10.1016/j.appet.2021.105122, PMID: 33453336 PMC9755826

[60] Waterschoot J Morbée S Vermote B Brenning K Flamant N Vansteenkiste M . Emotion regulation in times of COVID-19: a person-centered approach based on self-determination theory. Curr Psychol. (2023) 42:20211–25. doi: 10.1007/s12144-021-02623-5, PMID: 35039734 PMC8754525

[61] Xu F Huang L. Impacts of stress response and negative emotion on mental health of college students during the COVID-19 outbreak. Front Psychiatry. (2022) 12:784661. doi: 10.3389/fpsyt.2021.784661, PMID: 35350541 PMC8957802

[62] Ata RN Ludden AB Lally MM. The effects of gender and family, friend, and media influences on eating behaviors and body image during adolescence. J Youth Adolesc. (2007) 36:1024–37. doi: 10.1007/s10964-006-9159-x

[63] Shriver LH Dollar JM Calkins SD Keane SP Shanahan L Wideman L. Emotional eating in adolescence: effects of emotion regulation, weight status and negative body image. Nutrients. (2021) 13:79. doi: 10.3390/nu13010079 PMC7824438 33383717

[64] Bemanian M Mæland S Blomhoff R Rabben ÅK Arnesen EK Skogen JC . Emotional eating in relation to worries and psychological distress amid the COVID-19 pandemic: a population-based survey on adults in Norway. Int J Environ Res Public Health. (2021) 18:130. doi: 10.3390/ijerph18010130 PMC7795972 33375442

[65] Hadar-Shoval D Alon-Tirosh M Asraf K Tannous-Haddad L Tzischinsky O. Lifestyle changes, emotional eating, gender, and stress during COVID-19 lockdown. Nutrients. (2022) 14:3868. doi: 10.3390/nu14183868, PMID: 36145243 PMC9501635

[66] Page MJ McKenzie JE Bossuyt PM Boutron I Hoffmann TC Mulrow CD . Statement: an updated guideline for reporting systematic reviews. BMJ. (2020) 372:372. doi: 10.1136/bmj.n71, PMID: 33782057 PMC8005924

[67] Veritas Health Innovation. (2024). Covidence. Available online at: https://www.covidence.org/. (Accessed September 18, 2024)

[68] Joanna Briggs Institute. (2017). Checklist for analytical cross sectional studies. Available online at: https://jbi.global/sites/default/files/2019-05/JBI_Critical_Appraisal-Checklist_for_Analytical_Cross_Sectional_Studies2017_0.pdf (Accessed October 9, 2025).

[69] Ma LL Wang YY Yang ZH Huang D Weng H Zeng XT. Methodological quality (risk of bias) assessment tools for primary and secondary medical studies: What are they and which is better? Mil Med Res. (2020) 7:7. doi: 10.1186/s40779-020-00238-8, PMID: 32111253 PMC7049186

[70] R Core Team. (2021). R: a language and environment for statistical computing. Available online at: https://www.r-project.org/. (Accessed November 11, 2024)

[71] Viechtbauer W. Conducting meta-analyses in R with the metafor package. J Stat Softw. (2010) 36:1–48. doi: 10.18637/jss.v036.i03

[72] Stroup DF. Meta-analysis of observational studies in epidemiology. JAMA. (2000) 283:2008. doi: 10.1001/jama.283.15.2008 10789670

[73] Garaulet M Canteras M Morales E López-Guimera G Sánchez-Carracedo D Corbalán-Tutau MD. Validation of a questionnaire on emotional eating for use in cases of obesity: the Emotional Eater Questionnaire (EEQ). Nutr Hosp. (2012) 27:645–51. doi: 10.1590/S0212-16112012000200043 22732995

[74] Karlsson J Persson LO Sjöström L Sullivan M. Psychometric properties and factor structure of the Three-Factor Eating Questionnaire (TFEQ) in obese men and women. Results from the Swedish obese subjects (SOS) study. Int J Obes. (2000) 24:1715–25. doi: 10.1038/sj.ijo.0801442 11126230

[75] Cappelleri JC Bushmakin AG Gerber RA Leidy NK Sexton CC Lowe MR . Psychometric analysis of the Three-Factor Eating Questionnaire-R21: results from a large diverse sample of obese and non-obese participants. Int J Obes. (2009) 33:611–20. doi: 10.1038/ijo.2009.74, PMID: 19399021

[76] Schlundt DG Hargreaves MK Buchowski MS. The Eating Behavior Patterns Questionnaire predicts dietary fat intake in African American women. J Am Diet Assoc. (2003) 103:338–45. doi: 10.1053/jada.2003.50049, PMID: 12616256

[77] van Strien T Frijters JER Bergers GPA Defares PB. The Dutch Eating Behavior Questionnaire (DEBQ) for assessment of restrained, emotional and external eating behavior. Int J Eat Disord. (1986) 5:295–315. doi: 10.1002/1098-108X(198602)5:2<>3.0.CO;2-T

[78] Masheb RM Grilo CM. Emotional overeating and its associations with eating disorder psychopathology among overweight patients with Binge eating disorder. Int J Eat Disord. (2006) 39:141–6. doi: 10.1002/eat.20221, PMID: 16231349

[79] Vila-Marti A Ramírez-Contreras C Apolinar-Jiménez E Rojas-Cárdenas P Valera-Gran D Almendra-Pegueros R . Factors associated with dietary restriction and emotional and uncontrolled eating in adults from Spanish-speaking countries during the COVID-19 confinement: results of the CoV-Eat Project. Nutrients. (2022) 14:4866. doi: 10.3390/nu14224866, PMID: 36432551 PMC9693495

[80] Gearhardt AN Corbin WR Brownell KD. Preliminary validation of the Yale Food Addiction Scale. Appetite. (2009) 52:430–6. doi: 10.1016/j.appet.2008.12.003, PMID: 19121351

[81] Tazeoglu A Bozdogan FBK Idiz C. Evaluation of osmaniye korkut ata university students’ eating behaviors during the quarantine period during the COVID-19 pandemic period. Nutr Clin Diet Hosp. (2021) 41:86–93. doi: 10.12873/412tazeoglu

[82] Özcan BA Yeşilkaya B. Adverse effect of emotional eating developed during the COVID-19 pandemic on healthy nutrition, a vicious circle: a cross-sectional descriptive study. Rev Esp Nutr Hum Diet. (2021):25. doi: 10.14306/renhyd.25.S2.1144

[83] Carpio-Arias TV Piedra-Andrade JS Nicolalde-Cifuentes TM Padilla-Samaniego MV Tapia-Veloz EC Vinueza-Veloz MF. Mobility restrictions and mental health among young adults during the COVID-19 pandemic in Ecuador. Gac Sanit. (2022) 36:512–9. doi: 10.1016/j.gaceta.2021.12.008, PMID: 35339310 PMC8823959

[84] Hamzaid NH Gumisi ZGR Ahmad Helme SK Azmi N Shahril MR. Lifestyle and psychological factors affecting eating habits and physical activity among government servants in the states with the highest cumulative cases in Malaysia during the COVID-19 pandemic. Front Public Health. (2022) 10:816530. doi: 10.3389/fpubh.2022.816530, PMID: 35517647 PMC9062616

[85] Oliveira LA Della Lucia CM Rezende FAC Ferreira LG Anastácio LR Souza TC d M . Food craving and its associated factors during COVID-19 outbreak in Brazil. Am J Health Educ. (2022) 53:207–18. doi: 10.1080/19325037.2022.2071360

[86] Usubini AG Cattivelli R Varallo G Guerrini Usubini A Castelnuovo G Molinari E . The relationship between psychological distress during the second wave lockdown of COVID-19 and emotional eating in Italian young adults: the mediating role of emotional dysregulation. J Pers Med. (2021) 11:569. doi: 10.3390/jpm11060569, PMID: 34204480 PMC8235082

[87] Göbel P Sanlier N Yilmaz S Açikalin B Kocabaş Ş. The correlation between social media addiction and emotional eating during the COVID-19 quarantine period. Ecol Food Nutr. (2023) 62:60–74. doi: 10.1080/03670244.2023.2179044, PMID: 36803108

[88] Yoldas Ilktac H Savci C Cil Akinci A. Nutritional behavior during the COVID-19 pandemic: the association of fear and sleep quality with emotional eating. Eat Weight Disord. (2022) 27:2673–83. doi: 10.1007/s40519-022-01409-3, PMID: 35596840 PMC9123620

[89] Eşer Durmaz S Keser A Tunçer E. Effect of emotional eating and social media on nutritional behavior and obesity in university students who were receiving distance education due to the COVID-19 pandemic. J Public Health. (2023) 31:1645–54. doi: 10.1007/s10389-022-01735-x, PMID: 35891803 PMC9305038

[90] Carpio-Arias TV Solís Manzano AM Sandoval V Vinueza-Veloz AF Rodríguez Betancourt A Betancourt Ortíz SL . Relationship between perceived stress and emotional eating. A cross sectional study. Clin Nutr ESPEN. (2022) 49:314–8. doi: 10.1016/j.clnesp.2022.03.030, PMID: 35623832 PMC9817472

[91] Liboredo JC Anastácio LR Ferreira LG Oliveira LA Della Lucia CM. Quarantine during COVID-19 outbreak: eating behavior, perceived stress, and their independently associated factors in a Brazilian sample. Front Nutr. (2021) 8:704619. doi: 10.3389/fnut.2021.704619, PMID: 34381806 PMC8349978

[92] De Diego EH Zapatera B Frías J Gómez-Martínez S. Healthy habits in CSIC workers during COVID-19 lockdown in Spain. Arbor. (2022) 198:a681. doi: 10.3989/arbor.2022.806011

[93] Modrzejewska A Czepczor-Bernat K Modrzejewska J Matusik P. Eating motives and other factors predicting emotional overeating during COVID-19 in a sample of Polish adults. Nutrients. (2021) 13:1658. doi: 10.3390/nu13051658, PMID: 34068446 PMC8153582

[94] Karakose T Yirci R Basyigit H Kucukcakir A. Investigation of associations between the effects of COVID-19 fear on school administrators and nutrition and problematic eating behaviors. Prog Nutr. (2021) 23:e2021187. doi: 10.23751/pn.v23i2.11656

[95] Ederer DJ Lee SH Belay B Boutelle K Park S. Associations between comfort eating and weight change during the COVID-19 pandemic among U.S. adults. Human Nutr Metab. (2023) 33:200212. doi: 10.1016/j.hnm.2023.200212 PMC11010443 38618183

[96] Lo Moro G Bert F Catozzi D Scacchi A Siliquini R. Emotional eating and depression during the pandemic: QuarantEat, an Italian nationwide survey. Nutrition. (2022) 103-104:111825–104. doi: 10.1016/j.nut.2022.111825, PMID: 36183485

[97] Ricalde-Castillo Y Celis-Mendoza M Morales-García M Huancahuire-Vega S Calizaya-Milla YE Saintila J . Sociodemographic factors, mental health, and emotional eating associated with concern for COVID-19 contagion in nurses: a cross-sectional study. J Prim Care Community Health. (2023) 14:21501319231200400. doi: 10.1177/21501319231200400, PMID: 37731344 PMC10515584

[98] Elmacloǧlu F Emiroǧlu E Ülker MT Özyllmaz Klrcali B Oruç S. Evaluation of nutritional behaviour related to COVID-19. Public Health Nutr. (2021) 24:512–8. doi: 10.1017/S1368980020004140 33070798 PMC7737137

[99] Rahim HA Hoseini R Hoseini Z Abbas EN Kareem DA. Health-related factors of the Iraqi adult population during the 2020 COVID-19 pandemic: physical activity, eating behavior, quality of life, general health, and mood states cross-talk. BMC Public Health. (2023) 23:1046. doi: 10.1186/s12889-023-15898-z, PMID: 37264384 PMC10233189

[100] Serin E Koç MC. Examination of the eating behaviours and depression states of the university students who stay at home during the coronavirus pandemic in terms of different variables. Prog Nutr. (2020) 22:33–43. doi: 10.23751/pn.v22i1-S.9780

[101] Sumalla-Cano S Forbes-Hernández T Aparicio-Obregón S Crespo J Eléxpuru-Zabaleta M Gracia-Villar M . Changes in the lifestyle of the Spanish university population during confinement for COVID-19. Int J Environ Res Public Health. (2022) 19:2210. doi: 10.3390/ijerph19042210, PMID: 35206397 PMC8872173

[102] Di RL Gualtieri P Cinelli G Bigioni G Soldati L Attinà A . Psychological aspects and eating habits during COVID-19 home confinement: results of EHLC-COVID-19 Italian online survey. Nutrients. (2020) 12:2152. doi: 10.3390/nu12072152, PMID: 32707724 PMC7401000

[103] Wu C Hao M Liu X Yang D Liu B Yan W . The effects of body dissatisfaction and depression levels on the dietary habits of university students in southern China during COVID-19. Front Nutr. (2023) 10:1103724. doi: 10.3389/fnut.2023.1103724, PMID: 37599684 PMC10434794

[104] Smriti Sinha LG. Coping response to same stressors varies with gender. Natl J Physiol Pharm Pharmacol. (2018) 8:1053–6. doi: 10.5455/njppp.2018.8.0206921032018

[105] Braun-Lewensohn O. Coping strategies as mediators of the relationship between chronic exposure to missile attacks and stress reactions. J Child Adolesc Trauma. (2012) 5:315–26. doi: 10.1080/19361521.2012.719596

[106] United Nations. UN Secretary-General’s policy brief: the impact of COVID-19 on women. New York: UN Women Office Publishing (2020).

[107] Marcus SM Young EA Kerber KB Kornstein S Farabaugh AH Mitchell J . Gender differences in depression: findings from the STAR*D study. J Affect Disord. (2005) 87:141–50. doi: 10.1016/j.jad.2004.09.008, PMID: 15982748

[108] Alexandrino-Silva C Wang YP Carmen Viana M Bulhões RS Martins SS Andrade LH. Gender differences in symptomatic profiles of depression: results from the São Paulo megacity mental health survey. J Affect Disord. (2013) 147:355–64. doi: 10.1016/j.jad.2012.11.041, PMID: 23246363

[109] McLean CP Asnaani A Litz BT Hofmann SG. Gender differences in anxiety disorders: prevalence, course of illness, comorbidity and burden of illness. J Psychiatr Res. (2011) 45:1027–35. doi: 10.1016/j.jpsychires.2011.03.006, PMID: 21439576 PMC3135672

[110] Albert PR. Why is depression more prevalent in women? J Psychiatry Neurosci. (2015) 40:219–21. doi: 10.1503/jpn.150205, PMID: 26107348 PMC4478054

[111] Opanković A Latas M Jerotić S Ristić I Milovanović S. Gender differences in depression, anxiety, and stress during the first wave of the COVID-19 pandemic in Serbia—results from an online survey. Eur Psychiatry. (2021) 64:S270. doi: 10.1192/j.eurpsy.2021.724

[112] Queen C Pasupathy R Reine I. Multi-method analysis of gender differences in psychological distress among the elderly during COVID. Eur J Pub Health. (2022) 32:ckac130.207. doi: 10.1093/eurpub/ckac130.207

[113] Elran Barak R Shuval K Li Q Oetjen R Drope J Yaroch AL . Emotional eating in adults: the role of sociodemographics, lifestyle behaviors, and self-regulation—findings from a U.S. national study. Int J Environ Res Public Health. (2021) 18:1744. doi: 10.3390/ijerph18041744, PMID: 33670147 PMC7916829

[114] Lamichhane B Di Rosa E Braver TS. Delay of gratification dissociates cognitive control and valuation brain regions in healthy young adults. Neuropsychologia. (2022) 173:108303. doi: 10.1016/j.neuropsychologia.2022.108303, PMID: 35714970

[115] Glowacz F Schmits E. Psychological distress during the COVID-19 lockdown: the young adults most at risk. Psychiatry Res. (2020) 293:113486. doi: 10.1016/j.psychres.2020.113486, PMID: 33007682 PMC7518205

[116] Lee J Jeong HJ Kim S. Stress, anxiety, and depression among undergraduate students during the COVID-19 pandemic and their use of mental health services. Innov High Educ. (2021) 46:519–38. doi: 10.1007/s10755-021-09552-y, PMID: 33907351 PMC8062254

[117] Muennig P Lubetkin E Jia H Franks P. Gender and the burden of disease attributable to obesity. Am J Public Health. (2006) 96:1662–8. doi: 10.2105/AJPH.2005.068874, PMID: 16873748 PMC1551950

[118] Kapoor N Arora S Kalra S. Gender disparities in people living with obesity—an unchartered territory. J Midlife Health. (2021) 12:103–7. doi: 10.4103/jmh.jmh_48_21, PMID: 34526743 PMC8409720

[119] Van Blyderveen S Lafrance A Emond M Kosmerly S O’Connor M Chang F. Personality differences in the susceptibility to stress-eating: the influence of emotional control and impulsivity. Eat Behav. (2016) 23:76–81. doi: 10.1016/j.eatbeh.2016.07.009 27565374

[120] Yang H Zhou X Xie L Sun J. The effect of emotion regulation on emotional eating among undergraduate students in China: the chain mediating role of impulsivity and depressive symptoms. PLoS One. (2023) 18:e0280701. doi: 10.1371/journal.pone.0280701, PMID: 37319167 PMC10270349

[121] Chew HSJ Lopez V. Global impact of COVID-19 on weight and weight-related behaviors in the adult population: a scoping review. Int J Environ Res Public Health. (2021) 18:1876. doi: 10.3390/ijerph18041876 33671943 PMC7919006

[122] Neira C Godinho R Rincón F Mardones R Pedroso J. Consequences of the COVID-19 syndemic for nutritional health: a systematic review. Nutrients. (2021) 13:1168. doi: 10.3390/nu13041168, PMID: 33916087 PMC8065714

[123] Barcln-Güzeldere HK Devrim-Lanpir A. The association between body mass index, emotional eating and perceived stress during COVID-19 partial quarantine in healthy adults. Public Health Nutr. (2022) 25:43–50. doi: 10.1017/S1368980021002974 34261563 PMC8365042

[124] Jacob JS Panwar N. Effect of age and gender on dietary patterns, mindful eating, body image and confidence. BMC Psychol. (2023) 11:264. doi: 10.1186/s40359-023-01290-4, PMID: 37670398 PMC10478183

[125] Nashwan AJ Villar RC Al-Qudimat AR Kader N Alabdulla M Abujaber AA . Quality of life, sleep quality, depression, anxiety, stress, eating habits, and social bounds in nurses during the coronavirus disease 2019 pandemic in Qatar (The PROTECTOR study): a cross-sectional, comparative study. J Pers Med. (2021) 11:918. doi: 10.3390/jpm11090918, PMID: 34575695 PMC8468709

