# Supplementary material for: Feeding the feelings: gender differences in emotional eating during COVID-19: a systematic review and meta-analysis
Source: Front Nutr. 2025 Oct 27;12:1680872. doi: 10.3389/fnut.2025.1680872 (PMC12597742; doi:10.3389/fnut.2025.1680872)
Supplement: Supplementary file 1 [file Data_Sheet_1.zip › Supplementary Table S1.docx]

| Subgroup | K (studies) | Estimate  (Cohens d) | SE | 95% CI | p | R^2^ (%) | Interpretation |
| --- | --- | --- | --- | --- | --- | --- | --- |
| **Instrument type** |  |  |  |  |  | R² = 0% | No significant moderation (QM(6)=4.78, p=.573) |
| 1-Item-measure (ref.) | 1 | 0.36 | 0.27 | –0.16 to 0.88 | .175 |  | n.s. |
| DEBQ | 4 | 0.16 | 0.30 | –0.43 to 0.76 | .590 |  | n.s. |
| EBPQ | 1 | –0.06 | 0.42 | –0.88 to 0.76 | .882 |  | n.s. |
| EEQ | 5 | 0.04 | 0.30 | –0.54 to 0.62 | .887 |  | n.s. |
| EES | 2 | –0.03 | 0.33 | –0.68 to 0.61 | .915 |  | n.s. |
| EOQ-5 | 1 | –0.22 | 0.38 | –0.96 to 0.53 | .566 |  | n.s. |
| TFEQ-R18 | 2 | –0.31 | 0.33 | –0.96 to 0.33 | .336 |  | n.s. |
| **Region** |  |  |  |  |  | R² = 47.4% | Significant moderation (QM(6)=16.65, p=.011) |
| East Asia (ref.) | 1 | 0.40 | 0.20 | 0.01 to 0.78 | .042 |  | Significant: Women > Men |
| Europe | 4 | 0.07 | 0.22 | –0.36 to 0.50 | .746 |  | n.s. |
| Latin America | 2 | –0.19 | 0.24 | –0.66 to 0.28 | .422 |  | n.s. |
| Middle East | 6 | 0.02 | 0.21 | –0.40 to 0.44 | .940 |  | n.s. |
| Several regions | 1 | 0.05 | 0.28 | –0.49 to 0.59 | .851 |  | n.s. |
| Cross-regional Spanish-speaking | 1 | –0.73 | 0.27 | –1.26 to –0.20 | .007 |  | Significant: Men > Women (reversal) |
| South Asia | 1 | –0.10 | 0.33 | –0.74 to 0.54 | .765 |  | n.s. |
| **Sample type** |  |  |  |  |  | R² = 22.8% | Significant moderation (QM(1)=4.80, p=.028) |
| General population / professionals (ref.) | 9 | 0.22 | 0.08 | 0.06 to 0.38 | .008 |  | **Significant: Women > Men (moderate difference)** |
| Young adults / university students | 7 | 0.27 | 0.12 | 0.03 to 0.51 | .028 |  | **Significant: Women > Men (stronger difference)** |
| **Proportion women** |  | –0.11 | 0.10 | –0.30 to 0.09 | .273 | R² = 2.3% | n.s. |
| **Time period of data collection** |  |  |  |  |  | R² = 0% | n.s. (QM(4)=3.12, p=.54) |
| 2020–2021 (ref.) | 5 | 0.51 | 0.13 | 0.26 to 0.76 | <.001 |  | **Significant: Women > Men** |
| 2020 mid | 7 | –0.24 | 0.16 | –0.56 to 0.09 | .151 |  | n.s. |
| 2021 | 1 | –0.11 | 0.30 | –0.70 to 0.47 | .703 |  | n.s. |
| 2022 | 1 | –0.43 | 0.31 | –1.03 to 0.16 | .155 |  | n.s. |
| not specified | 2 | –0.18 | 0.25 | –0.66 to 0.31 | .475 |  | n.s. |
| **Sample size** |  | –0.00 | 0.00 | –0.00 to 0.00 | .311 | R² = 0% | n.s. |

**Supplementary Table S1. Subgroup and meta-regression results.**

Notes: Effect sizes are Cohen’s d (women − men); positive values indicate higher emotional eating in women. Reference categories: instrument = 1-item measure; region = East Asia; sample type = general population/professionals; time period = 2020–2021. Reported per subgroup: k (studies), estimate, SE, 95% CI, p-value, and model R². Significant omnibus tests: region QM(6)=16.65, p=.011; sample type QM(1)=4.80, p=.028; others n.s.
