# Supplementary material for: Feeding the feelings: gender differences in emotional eating during COVID-19: a systematic review and meta-analysis
Source: Front Nutr. 2025 Oct 27;12:1680872. doi: 10.3389/fnut.2025.1680872 (PMC12597742; doi:10.3389/fnut.2025.1680872)
Supplement: Supplementary file 1 [file Data_Sheet_1.zip › Supplementary Figure 1.pdf]

Feeding the feelings: Gender Differences in Emotional Eating During COVID-19: A Systematic Review and Meta-Analysis

Supplement

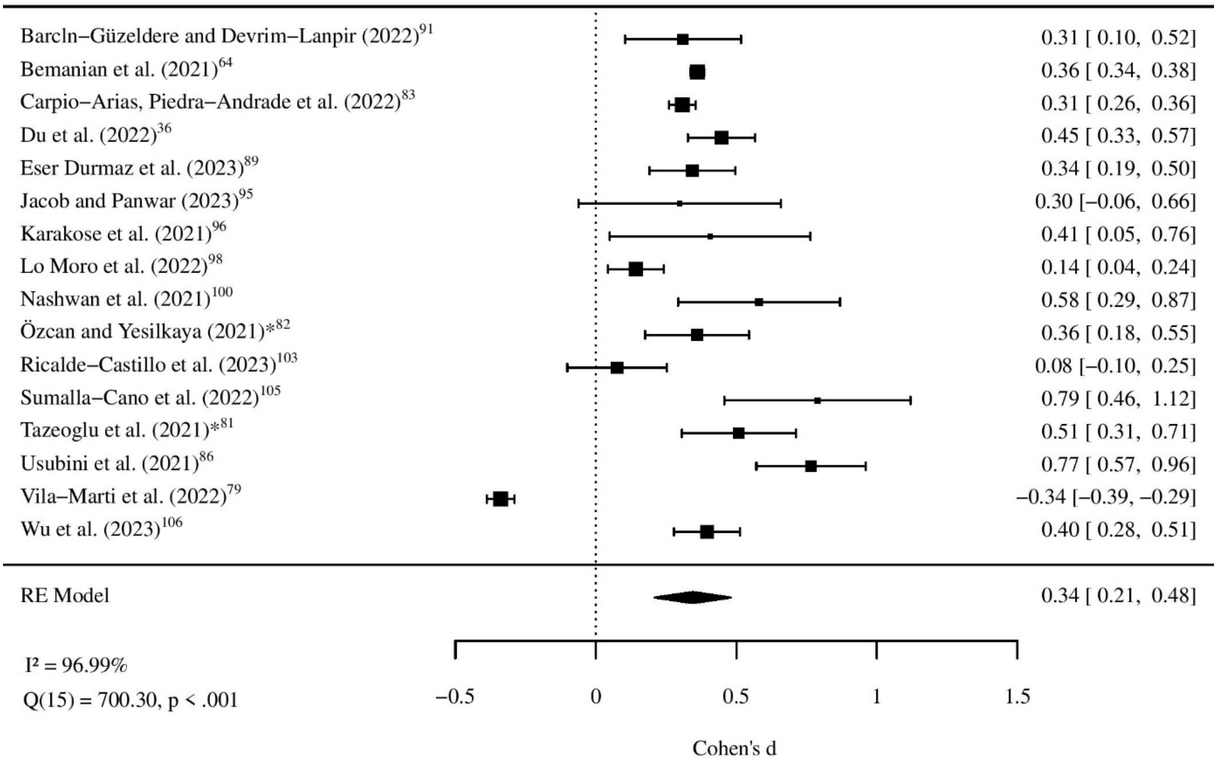

Supplemental Figure 1: Forest Plot of Initial Random-Effects Model

*Notes: All studies represent data collected during the COVID-19 pandemic, * indicates the second measurement point for Özcan and Yeşilkaya (2021) [82] and for Tazeoglu et al. (2021) [81].*
